# Apoptosis-resistant megakaryocytes produce large and hyperreactive platelets in response to radiation injury

**DOI:** 10.1186/s40779-023-00499-z

**Published:** 2023-12-19

**Authors:** Chang-Hong Du, Yi-Ding Wu, Ke Yang, Wei-Nian Liao, Li Ran, Chao-Nan Liu, Shu-Zhen Zhang, Kuan Yu, Jun Chen, Yong Quan, Mo Chen, Ming-Qiang Shen, Hong Tang, Shi-Lei Chen, Song Wang, Jing-Hong Zhao, Tian-Min Cheng, Jun-Ping Wang

**Affiliations:** 1 State Key Laboratory of Trauma, Burns and Combined Injury, Institute of Combined Injury, Chongqing Engineering Research Center for Nanomedicine, College of Preventive Medicine, Army Medical University, Chongqing, 400038 China; 2 Department of Nephrology, the Key Laboratory for the Prevention and Treatment of Chronic Kidney Disease of Chongqing, Kidney Center of PLA, Xinqiao Hospital, Army Medical University, Chongqing, 400037 China; 3 Frontier Medical Training Brigade, Army Medical University, Xinjiang, 831200 China

**Keywords:** Radiation injury, Thrombocytopenia, Platelet size, Platelet hyperreactivity, Megakaryocytes (MKs)

## Abstract

**Background:**

The essential roles of platelets in thrombosis have been well recognized. Unexpectedly, thrombosis is prevalent during thrombocytopenia induced by cytotoxicity of biological, physical and chemical origins, which could be suffered by military personnel and civilians during chemical, biological, radioactive, and nuclear events. Especially, thrombosis is considered a major cause of mortality from radiation injury-induced thrombocytopenia, while the underlying pathogenic mechanism remains elusive.

**Methods:**

A mouse model of radiation injury-induced thrombocytopenia was built by exposing mice to a sublethal dose of ionizing radiation (IR). The phenotypic and functional changes of platelets and megakaryocytes (MKs) were determined by a comprehensive set of in vitro and in vivo assays, including flow cytometry, flow chamber, histopathology, Western blotting, and chromatin immunoprecipitation, in combination with transcriptomic analysis. The molecular mechanism was investigated both in vitro and in vivo, and was consolidated using MK-specific knockout mice. The translational potential was evaluated using a human MK cell line and several pharmacological inhibitors.

**Results:**

In contrast to primitive MKs, mature MKs (mMKs) are intrinsically programmed to be apoptosis-resistant through reprogramming the Bcl-xL-BAX/BAK axis. Interestingly, mMKs undergo minority mitochondrial outer membrane permeabilization (MOMP) post IR, resulting in the activation of the cyclic GMP-AMP synthase-stimulator of IFN genes (cGAS-STING) pathway via the release of mitochondrial DNA. The subsequent interferon-β (IFN-β) response in mMKs upregulates a GTPase guanylate-binding protein 2 (GBP2) to produce large and hyperreactive platelets that favor thrombosis. Further, we unmask that autophagy restrains minority MOMP in mMKs post IR.

**Conclusions:**

Our study identifies that megakaryocytic mitochondria-cGAS/STING-IFN-β-GBP2 axis serves as a fundamental checkpoint that instructs the size and function of platelets upon radiation injury and can be harnessed to treat platelet pathologies.

**Supplementary Information:**

The online version contains supplementary material available at 10.1186/s40779-023-00499-z.

## Background

Thrombocytopenia, such as that occurrs in patients with inherited/acquired thrombocytopenia, microbial infection, radiotherapy, or chemotherapy [1], as well as in military personnel and civilians that have encountered chemical, biological, radioactive, and nuclear events [2], will significantly increase hemorrhagic risk. However, spontaneous hemorrhage does not always happen in mammals with thrombocytopenia [3]. Especially, in some cases such as ionizing radiation (IR) exposure [4–6], chemotherapy [7], and microbial infections including coronavirus disease 2019 [8], and gram-negative bacterial septicemia [9, 10], thrombocytopenia is unexpectedly associated with increased thrombotic risk, against the background of a hemorrhagic tendency. Meanwhile, thrombosis is increasingly being considered a major cause of mortality from thrombocytopenia [1], especially from that induced by radiation injury [6]. These lines of evidence imply that other yet to be defined factors such as the alteration of platelet function may be competent to maintain hemostasis or even favor thrombosis, unmasking which could open new opportunities to treat platelet disorders.

Platelets are produced by megakaryocytes (MKs) through a process termed thrombopoiesis [11]. The quantitative and qualitative properties of platelets are crucial determinants of their hemostatic capacity [12]. Albeit the regulatory mechanisms of platelet number and size have been elucidated separately, critical questions remain regarding the interplay of platelet number and size in hemostasis. An inverse relationship between platelet number and size in physiopathological conditions especially in thrombocytopenia has been frequently reported. It is generally thought that the increased thrombopoiesis evoked by thrombocytopenia generates young and large platelets [13]. However, this rule does not apply to some types of inherited or acquired thrombocytopenia as well as to increased thrombopoiesis following thrombopoietin (TPO) treatment [13, 14], implying that platelet size is not simply a reflection of platelet age but instead is tightly regulated during thrombopoiesis. Besides, platelet size is thought to be positively associated with platelet reactivity [15], inferring that platelet enlargement may be a compensation for the weakened hemostasis during thrombocytopenia. Unfortunately, the mechanisms that regulate platelet size and reactivity, especially in the context of thrombocytopenia are largely unknown.

In this study, using a mouse model of radiation injury-induced thrombocytopenia, we set out to explore the interplay of platelet number, size and reactivity as well as the underlying molecular basis. To evaluate the translational potential of this study, we also consolidated our findings by using a human MK cell line and several pharmacological inhibitors. This study provides novel insights into thrombopoiesis regulation, which has important implications for the pathogenesis and treatment of platelet disorders.

## Methods

### Animals

C57BL/6-Tg (Pf4-icre) Q3Rsko/J (*Pf4-Cre*) mice were purchased from the Jackson Laboratory (Bar Harbor, ME, USA). B6;129-Tmem173^tm1(flox)Smoc^ (*Sting*^*fl*^) mice were purchased from Shanghai Model Organisms Center, Inc. (Shanghai, China). C57BL/6J-Ifnar1^em1(flox)Cya^ (*Ifnar1*^*fl*^) mice were purchased from Cyagen Biosciences (Guangzhou, China). *Sting*^*cko*^ and *Ifnar1*^*cko*^ mice were generated by crossing *Pf4-Cre* mice with *Sting*^*fl*^ mice and *Ifnar1*^*fl*^ mice, respectively. Male *Sting*^*cko*^ (*n* = 22) and *Ifnar1*^*cko*^ (*n* = 17) mice as well as age-matched (8–10 weeks of age) *Sting*^*fl*^ (*n* = 25) and *Ifnar1*^*fl*^ (*n* = 20) mice were used for experiments. These mice were randomly assigned to the control group and IR group (*n* = 4–5 for each group as indicated in the figure legends). GFP-LC3 transgenic mice were kindly provided by professor Dengqun Liu (University of Electronic Science and Technology of China, Sichuan, China). A total of 16 GFP-LC3 mice were randomly assigned to the control group and IR group (*n* = 4 for each group). Wild-type C57BL/6 mice were purchased from Beijing HFK Bioscience Co., Ltd. (Beijing, China). A total of 388 C57BL/6 mice were randomly assigned to the control group and IR group (*n* = 3–10 for each group as indicated in the figure legends). For ABT-737 (MedChem Express, Monmouth Junction, NJ, USA) treatment, C57BL/6 mice were randomly assigned to the ABT-737 group and vehicle group (*n* = 5–10 for each group as indicated in the figure legends), and were respectively administered with a dose of 30 mg/kg ABT-737 or an equal volume of vehicle by intraperitoneal injection immediately post IR. For hydroxychloroquine (HCQ; MedChem Express) treatment, C57BL/6 mice were randomly assigned to the HCQ group and vehicle group (*n* = 5–6 for each group as indicated in the figure legends), and were respectively administered with a dose of 20 mg/kg HCQ or an equal volume of vehicle by intraperitoneal injection immediately post IR. The mice were kept in a pathogen-free facility at the Army Medical University under consistent environmental conditions, including a humidity level of (50 ± 5)%, a temperature of (24 ± 1) °C, a 12 h light/dark cycle, and free access to food and water. Animal experiments performed were approved by the Animal Care Committee of the Army Medical University and conducted according to the institutional guidelines (AMUWEC2019092).

### Cells isolation and culture

Meg-01 cells were grown in suspension using RPMI 1640 medium supplemented with 10% fetal bovine serum (all Thermo Fisher Scientific, Carlsbad, CA, USA). For ABT-737, BAI1, interferon-β (IFN-β), or chloroquine (CQ) treatment, Meg-01 cultures were incubated with 20 μmol/L ABT-737 (MedChem Express), 2 μmol/L BAI1 (Selleck Chemicals, Houston, TX, USA), 10^3^ U/ml recombinant human IFN-β (PeproTech, Rocky Hill, NJ, USA), or 20 μmol/L CQ (MedChem Express), respectively. Meg-01 viability was determined using a Cell Counting Kit-8 (MedChem Express).

Human cord blood-derived MKs were cultured as we previously reported [16]. Human cord blood samples were collected from the newborn boys and informed consent was obtained from the mother. CD34^+^ cells were isolated using an EasySep™ Human CD34 Positive Selection Kit II (StemCell Technologies, Vancouver, BC, Canada) according to the manufacturer’s instructions. Then, CD34^+^ cells were seeded in a 24-well plate and cultured in serum-free medium (StemSpan SFEM; Stem Cell Technologies) supplemented with 1% penicillin/streptomycin and 20 ng/ml recombinant human TPO (Peprotech) to allow for differentiation into MKs. Half of the media volume was changed every 3 d. Once the culture reached > 90% confluency, cultured cells in a well were split into 2–3 wells. The cells at 7, 10 and 13 d post TPO addition are generally recognized as MK progenitors (MkPs), differentiating MKs and mature MKs (mMKs), respectively [16]. The study protocol was approved by the Ethics Committee of Xinqiao Hospital (2018-006-01), and the study was carried out in accordance with the Declaration of Helsinki.

### Irradiation

Mice and cells received a single dose of 5 Gy γ-ray (total body irradiation) in a unilateral radiation field in the ^60^Co γ-radiation facility (Irradiation Center, Army Medical University, Chongqing, China). The dose rate was 92.8–95.5 cGy/min. The mice and cells were irradiated at room temperature in well-ventilated acrylic plastic boxes and polystyrene tubes, respectively. The boxes and tubes were placed 1.02 m away from the center of the ^60^Co source and within the 95% isodose area in the irradiator to ensure uniform delivery to multiple samples. Control mice and cells were sham-irradiated in similar conditions. For dosimetry in the position of the mice and cells, a PTW UNIDOS webline dosimeter system (Model T10002-20905, PTW, Freiburg, Germany) was used, which stopped the irradiation when a pre-defined dose value was reached. The calibration was performed by the National Institute of Metrology (Beijing, China) and was traceable to national standards.

### Platelet isolation and activation

After anesthesia, mouse blood was collected from the inferior vena cava with anticoagulant acid-citrate dextrose (ACD, 65 mmol/L trisodium citrate, 70 mmol/L citric acid, 100 mmol/L dextrose, pH 6.5). For washed platelets, the whole blood was centrifuged at 100 g for 15 min to obtain platelet-rich plasma, which was then centrifuged at 1300 g for 5 min in the presence of 1 μmol/L prostaglandin E1. Platelets were resuspended in modified Tyrode’s buffer (137 mmol/L NaCl, 2.7 mmol/L KCl, 12 mmol/L NaHCO_3_, 0.4 mmol/L NaH_2_PO_4_, 5 mmol/L HEPES, 5.6 mmol/L glucose, 0.35% bovine serum albumin, pH 7.4) at a density of 2 × 10^8^ platelets/ml to test their reactivity as we previously reported [17]. Briefly, the washed platelets were stimulated with thrombin (0.05 U/ml) in the presence of 1 mmol/L CaCl_2_, and stained with anti P-selectin-PE (eBioscience) or anti-activated integrin GPIIb/IIIa (JON/A-PE; Emfret Analytics, Eibelstadt, Germany) and anti-CD41-APC (eBioscience) for 15 min at room temperature (25 ± 5) °C. The reactions were stopped by the addition of 400 μl modified Tyrode’s buffer and analyzed using a FACSverse flow cytometer within 30 min. Platelets were selected based on forward scatter (FSC) and side scatter characteristics and CD41^+^ platelets were selected for analysis. The antibodies used were listed in Additional file 1: Table S1.

### Platelet-like particles (PLPs) analysis

PLPs were isolated from Meg-01 cultures as previously reported [18]. Briefly, PLPs were isolated from Meg-01 cultures by centrifugation at 200 g for 15 min. The precipitates were discarded and the supernatant was centrifuged at 750 g for 15 min. Then, the supernatants were separated and further centrifuged at 1600 g for 15 min, and precipitates containing PLPs were obtained for size and reactivity analysis.

### Platelet index determination

To determine platelet indices, 20 μl peripheral blood was collected from the tail vein of mice and diluted in 180 μl 1% ethylenediamine tetraacetic acid (EDTA) solution. Platelet count, mean platelet volume (MPV), platelet-larger cell ratio and platelet size deviation width were analyzed automatically by a Sysmex XT-2000i hematology analyzer (Sysmex Corporation, Kobe, Japan).

### Ex vivo flow chamber assay

Whole blood from control mice was centrifuged at 100 g for 10 min to obtain platelet-free blood cells and platelet-rich plasma. Platelet-free plasma was obtained from platelet-rich plasma by a second centrifugation step (600 g, 10 min). Then, the platelet-free blood cells were washed and mixed with platelet-free plasma to prepare platelet-free whole blood. Washed platelets (1 × 10^9^) pooled from irradiated mice or control mice were added into the platelet-free whole blood of control mice as prepared above. Subsequently, a flow chamber assay was performed as we previously reported [17]. Briefly, blood was incubated with rhodamine-6G (50 μl 0.05% per ml blood; Sigma-Aldrich, St. Louis, MO, USA) for 10 min at 37 °C. The whole blood was then perfused over microcapillary glass tubes coated overnight with type I collagen (150 μg/ml) or bovine serum albumin (background control) at a controlled shear rate (1800 s^−1^) using a syringe pump for 3 min. Adherent platelets and aggregates in the tubes were washed and micrographs of adhered platelets were acquired using a fluorescent microscope. Flow chamber surface coverage by the thrombus was measured as the area of platelet adhesion on collagen using ImageJ.

### Platelet-monocyte aggregate (PMA) assay

To detect PMAs, ACD-whole blood was treated with 1 ml red cell lysis buffer and carefully washed with phosphate‐buffered saline (PBS). Then, cells were stained with monoclonal antibodies against CD45, CD115, Gr-1, and CD41 (all eBioscience) for 30 min on ice. The cells were washed and resuspended in a Flow Cytometry Staining Buffer and run on a FACSverse flow cytometer. Viable cells were selected based on FSC and side scatter characteristics, and CD45^+^ leukocytes were selected for further analysis. PMAs were identified as CD115^+^Gr-1^high^ (Ly6-C^high^) and CD41^+^. The antibodies used were listed in Additional file 1: Table S1.

### Platelet-derived microparticle (PMP) detection

PMPs were detected as we previously reported [17]. Briefly, platelet-free plasma was prepared using serial centrifugations, diluted with annexin V binding buffer and incubated with antibodies to annexin V and CD41 (all eBioscience). PMPs were identified as particles that were less than 1 µm in size and stained positive for CD41. Activated PMPs were identified as PMPs that stained positive for annexin V. The antibodies used were listed in Additional file 1: Table S1.

### Platelet age analysis

ACD-whole blood (1 μl) was diluted in 10 μl modified Tyrode’s buffer. Then, 50 μl thiazole orange (Sigma-Aldrich) staining solution (0.1 mg/ml in modified Tyrode’s buffer) was added in the presence of an anti-CD41 antibody. Reactions were incubated in the dark at room temperature for 15 min. The antibodies used were listed in Additional file 1: Table S1.

### Mouse mMKs isolation

Bone marrow cells (BMCs) were flushed from the femur and tibia, and red blood cells were lysed using a red cell lysis buffer. Mouse mMKs were obtained by continuously enriching CD41^+^ cells and CD42d^+^ cells using an EasySep™ Release Mouse Biotin Positive Selection Kit and an EasySep™ Release Mouse Phycoerythrin Positive Selection Kit (all StemCell Technologies) with biotin-labeled anti-CD41 and Phycoerythrin-labeled anti-CD42d antibodies according to the manufacturer’s instructions. The antibodies used were listed in Additional file 1: Table S1.

### Microarray

RNA was extracted from sorted mMKs using a TRIzol Plus reagent (TaKaRa, Shiga, Japan). RNA quality and quantity were examined by an Agilent 4200 TapeStation System. cDNA was synthesized using a cDNA synthesis kit (Affymetrix, Inc., Santa Clara, CA, USA) and labeled using a GeneChip® WT PLUS Reagent Kit (Affymetrix, Inc.). Then, the labeled cDNA was hybridized to the mouse Gene chip at 45 °C for 16 h. After washing with wash solution A, wash solution B and deionized water, the Gene chip was stained with Cocktail 1 and Cocktail 2. The GeneChip® Scanner 3000 7G (Affymetrix Inc.) and the AGCC Scan Control software (Affymetrix Inc.) were used for data analysis. Microarray hybridization was carried out by Shanghai GMINIX Biotech Limited Company (Shanghai, China) using the GeneChip® Mouse Gene 1.0 ST Array (Affymetrix Inc.). Gene set enrichment analysis was performed using Molecular Signatures Database v.2022.1 (http://software.broadinstitute.org/gsea/msigdb).

### Flow cytometry

BMCs were flushed from the femur and tibia and red blood cells were lysed using a red cell lysis buffer (StemCell Technologies). For hematopoietic cell phenotypic analysis, a lineage cocktail was used, including CD3, CD11b, Gr-1, B220 and Ter-119 (eBioscience, San Diego, CA, USA). Mouse MkP (Lineage^−^Sca1^−^c-Kit^+^CD41^+^CD150^+^), mMK (CD41^+^CD42d^+^), macrophage (CD45^+^CD80^+^CD11b^+^), and dendritic cell (DC, CD45^+^CD11c^+^) were analyzed using monoclonal antibodies as indicated. Cells were sorted using a FACSAria III or analyzed using a FACSverse flow cytometer (all BD Biosciences, San Jose, CA, USA). Data analysis was performed using FlowJo software (Treestar Inc., San Carlos, CA, USA). The expression of intracellular phosphorylated protein was detected as we previously reported [19]. Briefly, pre-stained BMCs were first fixed with an Intracellular Fixation Buffer (eBioscience) at room temperature for 20 min. Then, the cells were resuspended with 1 ml ice-cold methanol and incubated on ice for 30 min. After carefully washing, the cells were resuspended with Flow Cytometry Staining Buffer (eBioscience) in the presence of anti-p-TANK-binding kinase 1 (TBK1), anti-p-interferon regulatory factor 3 (IRF3) (all Cell Signaling Technology) or anti-p-signal transducer and activator of transcription 1 (STAT1) (Biolegend, San Diego, CA, USA) for another 30 min at room temperature, followed by flow cytometric analysis. To detect cytoplasmic protein expression, pre-stained BMCs were fixed with Intracellular Fixation buffer (eBioscience) at room temperature for 30 min and subsequently permeabilized with a permeabilization buffer (eBioscience) in the presence of anti-active caspase-3 (Cell Signaling Technology), anti-Bcl-xL, anti-MCL1, anti-BAX, anti-BAK, anti-guanylate-binding protein 2 (GBP2) antibodies (all Thermo Fisher Scientific), anti-cytochrome C (Cyt-C) (Biolegend), or anti-IFN-β (Abcam) at room temperature for another 30 min. Finally, the cells were stained with fluorescent dye conjugated secondary antibodies (Thermo Fisher Scientific) and analyzed by flow cytometry. The antibodies used were listed in Additional file 1: Table S1.

### Apoptosis analysis

Apoptosis analysis was performed using an Annexin V-eFluor 450 Apoptosis Detection Kit (eBioscience). Pre-stained BMCs were resuspended in a suitable volume of 1 × Binding Buffer. Then, Annexin V-eFluor 450 antibody was added and incubated for 10 min at room temperature. After washing with 1 × Binding Buffer, 7-amino-actinomycin D staining solution was added and the cells were immediately analyzed by flow cytometry. The antibodies used were listed in Additional file 1: Table S1.

### Mitochondrial membrane potential (MMP) analysis

The MMP of MKs was analyzed using a tetramethylrhodamine ethyl ester dye (Thermo Fisher Scientific) according to the manufacturer’s instructions. Briefly, pre-stained BMCs were suspended in 1 ml prewarmed (37 °C) Flow Cytometry Staining Buffer with 100 nmol/L tetramethylrhodamine ethyl ester, together with 50 μmol/L verapamil (Sigma-Aldrich). After being incubated at 37 °C for 30 min, cells were washed twice and immediately analyzed by a FACSverse flow cytometer. To induce complete MMP collapse, pre-stained BMCs were treated with 50 μmol/L carbonyl cyanide m-chlorophenylhydrazine (Thermo Fisher Scientific) for 5 min.

### Mitochondrial Cyt-C determination

BMCs were stained with indicated surface markers and carefully washed. One milliliter of Foxp3 Fixation/Permeabilization working solution (eBioscience) was added to the resuspended cells and incubated at room temperature for 30 min. Then, the cells were permeabilized with Permeabilization buffer in the presence of anti-Cyt-C (Biolegend) at room temperature for 30 min, followed by flow cytometric analysis with a FACSverse flow cytometer. The antibodies used were listed in Additional file 1: Table S1.

### Cytosolic mitochondrial DNA (mtDNA) measurement

Meg-01 cells were resuspended in 170 μl of digitonin buffer containing 150 mmol/L NaCl, 50 mmol/L HEPES (pH 7.4), and 25 µg/ml digitonin (Sigma-Aldrich). The homogenates were incubated on a rotator for 10 min at room temperature, followed by centrifugation at 16,000 g for 25 min at 4 °C. Then, the supernatant was used for quantitative polymerase chain reaction (qPCR) to detect cytosolic mtDNA and nuclear DNA (nDNA). The pellet was resuspended in 340 μl of lysis buffer containing 5 mmol/L EDTA and proteinase K (Sigma-Aldrich) and incubated at 55 °C overnight. The digested pellet was diluted with water and heated at 95 °C for 20 min to inactivate proteinase K, and the sample was used for qPCR to detect total mtDNA and nDNA. The cytosolic mtDNA/nDNA in the supernatant was normalized to the total mtDNA/nDNA in the pellet for each sample. The primer sequences used were listed in Additional file 1: Table S2.

### RNA interference

RNA interference was conducted as we previously reported [16]. Briefly, Meg-01 cells were washed with ice-cold PBS and approximately 5 × 10^6^ cells were resuspended in 0.5 ml of Opti-MEM® Medium (Thermo Fisher Scientific), mixed with 20 nmol siRNA of cyclic GMP-AMP synthase (cGAS), stimulator of IFN genes (STING), IFN‐α/β receptor 1 and GBP2 (all Genepharma, Shanghai, China). Then, cells were electroporated at 0.3 kV and 500 μF using a Bio-Rad Gene Pulser Xcell electroporation system (Bio-Rad). Twenty-four hours post electroporation, cells were exposed to IR or treated with IFN-β. The siRNA sequences used are listed in Additional file 1: Table S3.

### Cell transfection

To detect autophagy in vitro, Meg-01 cells were transfected with HBLV-mCherry-GFP-LC3-PURO lentivirus (Hanbio Biotechnology Co. Ltd, Shanghai, China) as we previously reported [20]. Cells were infected with lentivirus at a multiplicity of infection of 20 in the presence of 8 µg/ml polybrene. mCherry-GFP-LC3 expression was validated by fluorescence microscope 48 h post-transduction and 4 μg/ml puromycin was added to select cells with stable mCherry-GFP-LC3 expression.

### Flag-cGAS cloning and chromatin immunoprecipitation

The identity of cGAS-bound DNA was determined as we previously reported [19]. Briefly, 3 × Flag-tagged human cGAS was sub-cloned into a pCDH-CMV-MCS-EF1-GFP + Puro lentiviral vector (Genecreate, Wuhan, China) and Meg-01 cells were stably transfected with this virus. At 1 day post IR (dpi), the stably-transfected Meg-01 cells were fixed with 1% formaldehyde (Sigma-Aldrich) for 10 min. After washing three times with ice-cold PBS, the cells were disrupted by sodium dodecyl sulfate lysis buffer (Millipore, Billerica, MA, USA) and divided into two aliquots. Then, the cross-linked protein/DNA in one aliquot was immunoprecipitated using an anti-FLAG M2 antibody (Sigma-Aldrich). The purified DNA from whole-cell extracts and immunoprecipitated DNA were examined by qPCR using a GoTaq qPCR Master Mix (Promega) on a CFX96 Real-Time system (Bio-Rad). The DNA abundance of whole-cell extracts served as a normalization control for the immunoprecipitated DNA abundance. The antibody and primer sequences used were listed in Additional file 1: Tables S1, S2.

### ELISA

Bone marrow extracellular fluid was collected as we previously reported [21]. IFN-β levels in bone marrow extracellular fluid were measured in duplicate using a mouse IFN-β ELISA Kit (R&D Systems, Minneapolis, MN, USA) according to the manufacturer’s instructions. The optical density was measured using a Tecan Infinite 200® PRO microplate reader.

### Western blotting

Cells were homogenized in radio-immunoprecipitation assay buffer supplemented with Pierce™ EDTA-free Protease Inhibitor Tablets and Pierce™ Phosphatase Inhibitor Mini Tablets (all Thermo Fisher Scientific) for 30 min at 4 °C, and then centrifuged at 10,000 g for 15 min at 4 °C. The protein concentrations were determined by a bicinchoninic acid Protein Concentration Determination Kit (Thermo Fisher Scientific). The proteins were separated by sodium dodecyl sulfate polyacrylamide gel electrophoresis. Protein expression was determined using anti-p-STAT1, anti-STAT1, anti-TBK1, anti-IRF3, anti-p-TBK1, anti-p-IRF3, anti-GAPDH (all Cell Signaling Technology), anti-IFN-β, or anti-GBP2 (all Abcam, Cambridge, UK) antibodies at 4 °C overnight. Finally, membranes were incubated with appropriate secondary antibodies (Abcam) and scanned by a Bio-Rad ChemiDoc MP imager (Bio-Rad). The antibodies used were listed in Additional file 1: Table S1.

### qPCR

RNA was extracted using a TRIzol Plus reagent (TaKaRa). Then, mRNA was treated at 42 °C for 2 min with gDNA Eraser (TaKaRa), and reverse-transcribed into cDNA using a PrimeScript™ RT reagent Kit (TaKaRa) according to the manufacturer’s instructions. The mRNA expression of indicated genes was determined using a GoTaq® qPCR Master Mix (Promega, Madison, WI, USA) on a CFX96™ Real-Time system (Bio-Rad, Hercules, CA, USA). Data was normalized relative to GAPDH. The primer sequences used were listed in Additional file 1: Table S4.

### Immunofluorescence

Femurs were fixed in 4% formaldehyde and then decalcified in 10% EDTA-PBS buffer. After being embedded in paraffin, the prepared samples were cut into 5-μm-thick sections. Meg-01 cells were spun onto poly-L-lysine coating slides, fixed with 4% paraformaldehyde at room temperature for 10 min. For immunostaining, antigen retrieval was achieved by boiling in citrate buffer (Santa Cruz Biotechnology, Santa Cruz, CA, USA) above 95 °C for 30 min. Then, sections were blocked with goat serum (Santa Cruz Biotechnology) for 40 min at room temperature. After permeabilization with 0.25% Triton X-100 (Santa Cruz Biotechnology) for 10 min and blockade with goat serum for 40 min at room temperature, the slides were stained for p-STAT1, p-TBK1, p-IRF3 (all Cell Signaling Technology), and IFN-β (Abcam) antibodies at 4 °C overnight. To detect the colocalization of cGAS with DNA, the sections were stained for cGAS (Abcam) and DNA (Millipore) antibodies; Meg-01 cells were stained for cGAS (Santa Cruz Biotechnology) and DNA (Millipore) antibodies at 4 °C overnight. To detect autophagic clearance of mitochondria, the slides were stained for LC3 (Novus Biologicals) and TOM20 (Santa Cruz Biotechnology) antibodies. Then, the sections were stained with appropriate fluorescent dye conjugated secondary antibodies (Thermo Fisher Scientific). Finally, the sections were counterstained with 4′,6-diamidino-2-phenylindole and photographed under a Zeiss LSM780 NLO confocal microscope (Carl Zeiss, Jena, Germany). The antibodies used were listed in Additional file 1: Table S1.

### Statistical analysis

Statistical analysis was performed using Prism v9.3.1 (GraphPad Software, La Jolla, CA, USA). All results are presented as mean ± standard deviation. *n* represents the mouse number analyzed in each experiment, as described in the figure legends. Comparisons between the two groups were determined by a two-tailed unpaired Student’s *t*-test. Three groups were compared by a one-way analysis of variance followed by a Tukey–Kramer post hoc analysis. Kaplan–Meier curves and Log-rank tests were used for survival analysis. *P* < 0.05 was considered statistically significant.

## Results

### mMKs are resistant to IR and produce large platelets post IR

**Fig. 1:**
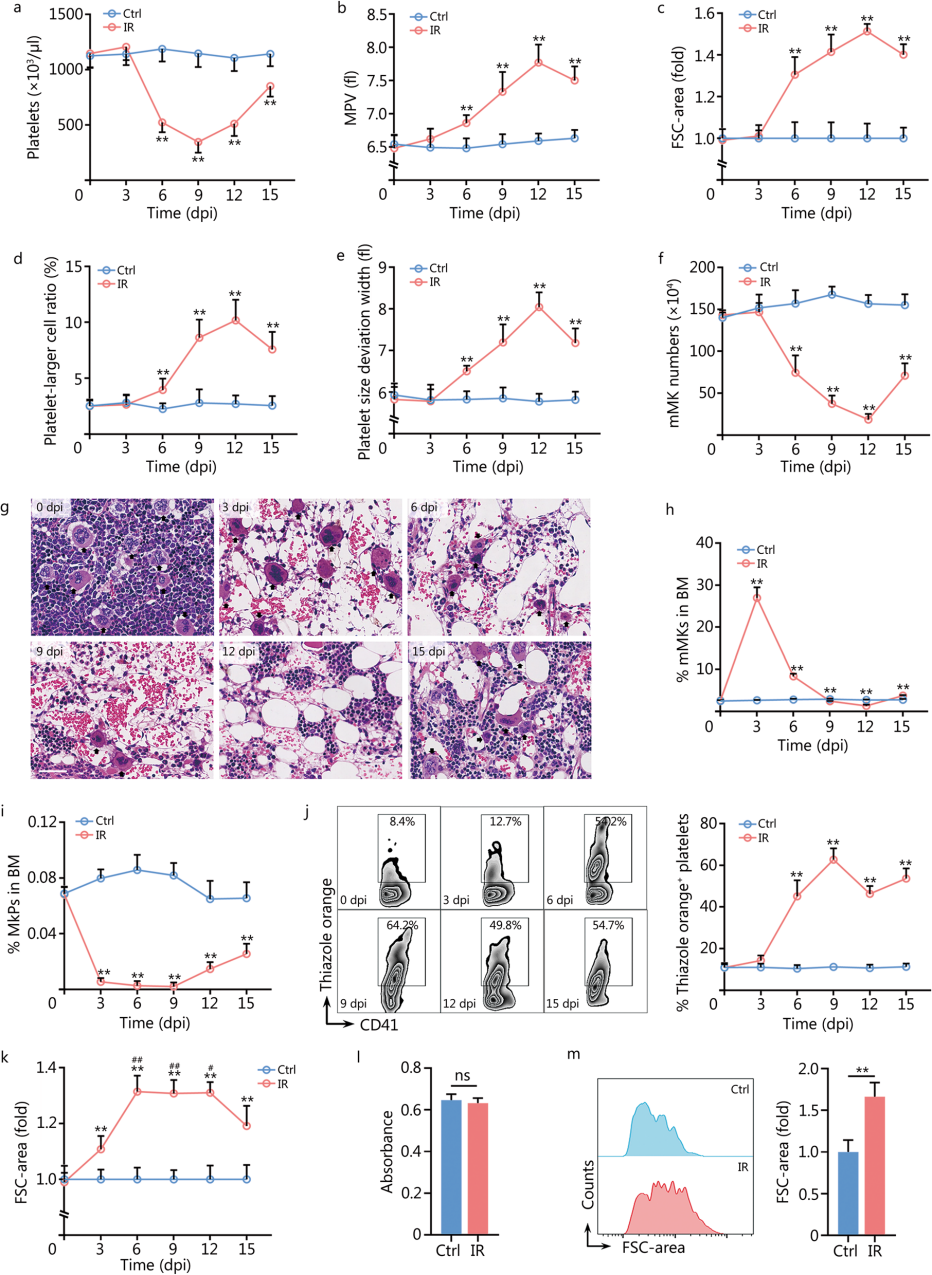
mMKs are resistant to IR and produce large platelets post IR. **a** Platelet counts in peripheral blood of mice at indicated dpi (*n* = 10). **b** MPV in peripheral blood of mice at indicated dpi (*n* = 10). **c** Flow cytometric quantification of FSC-area of platelets in peripheral blood of mice at indicated dpi (*n* = 6). **d, e** Platelet-larger cell ratio and platelet size deviation width in peripheral blood of mice at indicated dpi (*n* = 10). **f** mMK numbers in BM of mice at indicated dpi (*n* = 6). **g** Representative hematoxylin and eosin staining of longitudinal sections of femurs of mice at indicated dpi. Scale bar = 40 μm. **h** Frequency of mMKs in BM of mice at indicated dpi (*n* = 6). **i** Frequency of MkPs in BM of mice at indicated dpi (*n* = 6). **j** Flow cytometric analysis and quantification of the frequency of nascent (thiazole orange^+^) platelets in peripheral blood of mice at indicated dpi (*n* = 6). **k** Flow cytometric quantification of FSC-area of thiazole orange^+^ platelets in peripheral blood of mice at indicated dpi (*n* = 6). **l** Viability of Meg-01 cells at 3 dpi as determined by Cell Counting Kit-8 (*n* = 5). **m** Flow cytometric analysis and quantification of FSC-area of PLPs in Meg-01 cultures at 3 dpi (*n* = 5). Data represent mean ± standard deviation. ^**^P < 0.01, compared to Ctrl, two-tailed unpaired student’s *t*-test unless stated otherwise. ^#^*P* < 0.05, ^##^*P* < 0.01, compared to 15 dpi, one-way analysis of variance (**k**). ns non-significance, mMK mature megakaryocyte, Ctrl control, IR ionizing radiation, dpi day post IR, MPV mean platelet volume, FSC forward scatter, BM bone marrow, MkP megakaryocyte progenitor, PLP platelet-like particle

Initially, we examined platelet indices in mice after exposure to sublethal IR. Consistently, platelet numbers declined rapidly from the 3 dpi, reaching the nadir by 9 dpi (Fig. 1a). In contrast, platelet size, as determined by MPV (Fig. 1b) and FSC-area (Fig. 1c), gradually increased post IR, reaching the peak by 12 dpi. Parallelly, both platelet-larger cell ratio (Fig. 1d) and platelet size deviation width (Fig. 1e) were elevated, indicating a gradual accumulation of large platelets post IR. Subsequently, we analyzed the bone marrow MK compartment post IR. It was observed that mMKs were more resistant to IR as mMKs were scarcely eliminated by 3 dpi (Fig. 1f, g), unlike the vast majority of BMCs (Additional file 1: Fig. S1). As a result, the proportion of mMKs in bone marrow sharply increased by 3 dpi (Fig. 1h). However, mMKs began to rapidly decrease from 3 dpi and reached the minimum by 12 dpi (Fig. 1f–h), paralleling the sharp increase of platelet size post IR. On the contrary, MkPs were extremely sensitive to IR, with only very few MkPs surviving, and the generation of MkPs was constantly suppressed until 9 dpi (Fig. 1i). Meanwhile, although mMKs were newly generated by 15 dpi (Fig. 1f), the platelet size declined (Fig. 1b–e), suggesting that the platelets produced by surviving mMKs might be larger than those produced by newly-generated mMKs. To confirm this, the age profile of circulating platelets was examined by thiazole orange staining. A dramatic increase in the proportion of nascent platelets was observed from 3 dpi (Fig. 1j), indicating an active platelet production by surviving mMKs. Moreover, the nascent platelets produced during the period of 6–12 dpi were strikingly larger than those in control mice and those produced by newly-generated mMKs at 15 dpi (Fig. 1k). In vitro, Meg-01, a well-accepted and biologically relevant human mMK cell line [16, 18], also mostly survived IR (Fig. 1l) and produced large PLPs post IR (Fig. 1m). Given that the lifespan of murine platelets is normally 4–5 d [22] and IR does not directly affect platelet size [23], the gradual increase in platelet size post IR may result from a combination of continuous accumulation of large platelets produced by surviving mMKs and continuous depletion of pre-existing, relatively small platelets produced before IR.

### Large platelets produced post IR are inherently hyperreactive

**Fig. 2:**
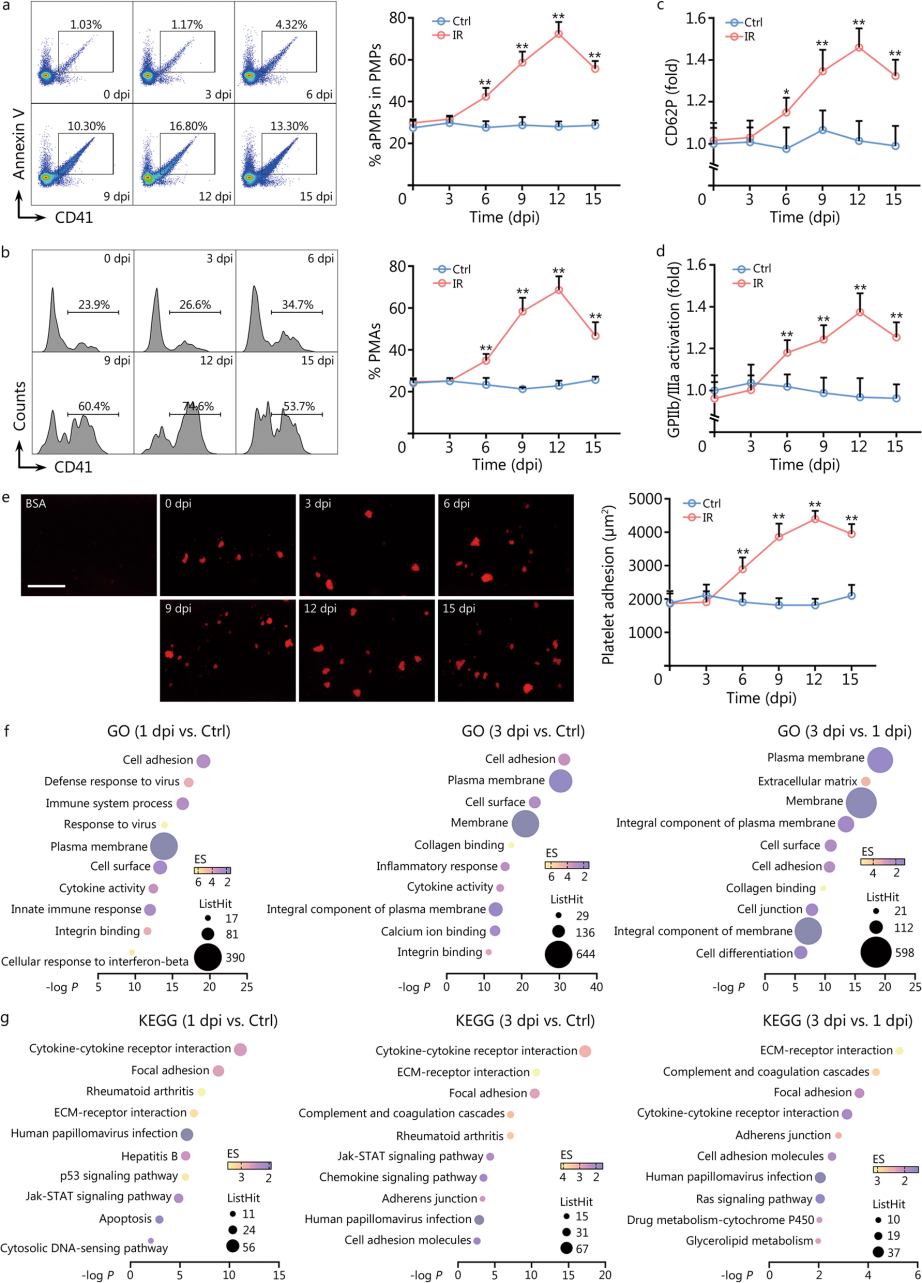
Large platelets produced post IR are inherently hyperreactive. Flow cytometric analysis and quantification of the frequency of PMPs (**a**) and PMAs (**b**) in peripheral blood of mice at indicated dpi (*n* = 5). Flow cytometric quantification of CD62P expression (**c**) and GPIIb/IIIa activation (**d**) on washed platelets in response to thrombin at indicated dpi (*n* = 5). **e** Representative images and quantification of thrombus formation on collagen-coated flow chambers under shear-flow conditions using platelets obtained from mice at indicated dpi (*n* = 5). Scale bar = 250 μm. **f, g** GO and KEGG enrichment analysis of top upregulated signaling pathways in BM mMKs of mice at 1 and 3 dpi. Data represent mean ± standard deviation. ^**^P < 0.01, compared to Ctrl. Two-tailed unpaired student’s *t*-test. Ctrl control, IR ionizing radiation, dpi day post IR, PMP platelet-derived microparticle, aPMP activated PMP, PMA platelet-monocyte aggregate, BSA bovine serum albumin, GO Gene Ontology, KEGG Kyoto Encyclopedia of Genes and Genomes, BM bone marrow, mMK mature megakaryocyte, ECM extracellular matrix, Jak Janus kinase, STAT signal transducer and activator of transcription

Platelet size is usually positively correlated with platelet reactivity [13]. Indeed, the frequencies of activated PMPs (Fig. 2a) and PMAs (Fig. 2b), two sensitive markers of platelet activation [17], were dramatically increased post IR, paralleling the change dynamics of platelet size. To accurately determine whether the hyperreactivity of circulating platelets post IR reflected properties intrinsic to platelets, we proceeded to examine the hemostatic function of platelets in vitro with the exclusion of the interference by platelet numbers and other cells such as leukocytes. P-selectin/CD62P surface expression and integrin GPIIb/IIIa activation are the hallmarks of platelet activation [17]. Washed platelets from IR mice exhibited dramatically increased fold changes of surface CD62P expression (Fig. 2c) and GPIIb/IIIa activation (Fig. 2d) in response to thrombin stimulation. Flow chamber assay with a constant number of platelets revealed that platelets from IR mice adhered faster to collagen and formed larger thrombi than those from control mice (Fig. 2e). Correspondingly, RNA-seq revealed that mMKs at 1 and 3 dpi exhibited a gradually enhanced platelet hyperreactivity signature that was manifested by robustly upregulated expression of adhesion molecules as well as collagen and integrin-binding molecules (Fig. 2f, g). Gene set enrichment analysis also detected significantly upregulated gene sets associated with platelet activation and aggregation in mMKs post IR (Additional file 1: Fig. S2a). As did results in vivo, the mRNA expression of adhesion acceptors (*VLA-6* and *GPVI*) and *CD62P* that are critical for platelet activation was significantly upregulated in Meg-01 post IR (Additional file 1: Fig. S2b). Meanwhile, PLPs from Meg-01 culture at 3 dpi were hyperreactive to thrombin as well (Additional file 1: Fig. S2c). Thus, the large platelets produced by surviving mMKs post IR are inherently hyperreactive.

### The inherently high pro-survival threshold confers radioresistance onto mMKs

**Fig. 3:**
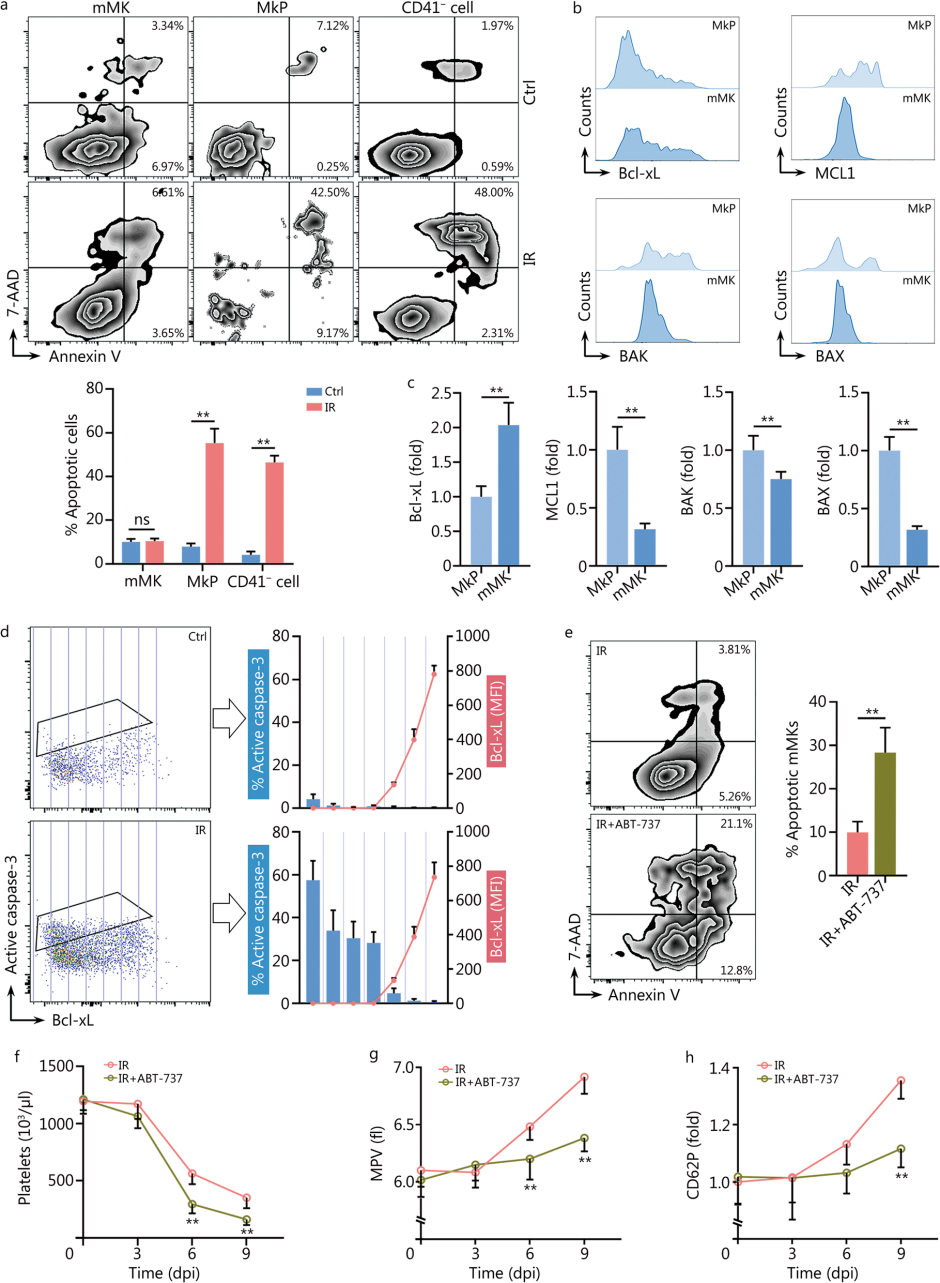
The inherently high pro-survival threshold confers radioresistance onto mMKs. **a** Flow cytometric analysis and quantification of MK and non-MK apoptosis in BM of mice at 1 dpi (*n* = 5). **b, c** Flow cytometric analysis and quantification of pro-survival (Bcl-xL and MCL1) and pro-apoptotic (BAX and BAK) Bcl-2 family protein expression in BM MKs of mice (*n* = 5). **d** Flow cytometric analysis and quantification of the relationship between Bcl-xL expression and apoptosis as reflected by active caspase-3 expression in BM CD41^+^ MKs of mice at 1 dpi (*n* = 5). **e** Flow cytometric analysis and quantification of mMK apoptosis in BM of mice with or without ABT-737 treatment at 1 dpi (*n* = 5). Platelet counts (**f**) and MPV (**g**) in peripheral blood of mice with or without ABT-737 treatment at indicated dpi (*n* = 6). **h** CD62P expression in response to thrombin on washed platelets from mice with or without ABT-737 treatment at indicated dpi (*n* = 5). Data represent mean ± standard deviation. ^**^P < 0.01, compared to Ctrl, MkP or IR as indicated. Two-tailed unpaired student’s *t*-test. ns non-significance, MK megakaryocyte, mMK mature MK, MkP MK progenitor, Ctrl control, IR ionizing radiation, dpi day post IR, 7-AAD 7-amino-actinomycin D, MFI mean fluorescence intensity, BM bone marrow, MPV mean platelet volume

The above results indicate that the alterations in platelet size and reactivity are mMK-autonomous. We next interrogated how mMKs survived and responded to IR. As known, hematopoietic cells are extremely sensitive to IR and will immediately undergo apoptosis post IR [24]. However, almost no mMKs underwent apoptosis compared with MkPs and other lineages post IR **(**Fig. 3a). Similarly, Meg-01 cells were also resistant to IR-induced apoptosis (Additional file 1: Fig. S3a). The mitochondrial apoptotic pathway plays a dominant role in IR-induced apoptosis. In this process, pro-apoptotic Bcl-2 family proteins either can be bound and sequestered by pro-survival Bcl-2 family proteins or, when these pro-survival Bcl-2 family proteins are saturated, can activate BAX/BAK to permeabilize mitochondrial outer membrane, an event termed mitochondrial outer membrane permeabilization (MOMP) that results in apoptosis through releasing mitochondrial factors including Cyt-C that activates caspases. Among the Bcl-2 family proteins, pro-survival Bcl-xL and pro-apoptotic BAX/BAK are mitochondrial outer membrane components that establish the apoptotic threshold of a cell [25]. Interestingly, a shift of the balance of pro-survival and pro-apoptotic Bcl-2 family molecules towards survival was observed during maturation of both murine (Fig. 3b, c**, **Additional file 1: Fig. S3b) and human (Additional file 1: Fig. S3c) MKs, with Bcl-xL being upregulated whereas BAX/BAK being downregulated. After IR, apoptosis occurred preferentially in MKs expressing lower Bcl-xL (Fig. 3d). Then, we lowered the pro-survival threshold using ABT-737, a Bcl-xL inhibitor. Strikingly, ABT-737 treatment significantly sensitized mMKs to IR-induced apoptosis both in vivo (Fig. 3e) and in vitro (Additional file 1: Fig. S3d). Consequently, both platelet counts (Fig. 3f) and size (Fig. 3g) significantly declined in mice with ABT-737 treatment post IR, accompanied by significantly decreased platelet reactivity (Fig. 3h). Meanwhile, ABT-737-treated mice were more sensitive to sublethal IR, as all mice died by 11 dpi (Additional file 1: Fig. S3e). In vitro, the size and reactivity of PLPs from ABT-737-treated Meg-01 cultures also significantly declined post IR (Additional file 1: Fig. S3f). Therefore, MK maturation is associated with an enhanced pro-survival threshold, thereby conferring radioresistance onto mMKs.

### Minority MOMP is triggered in mMKs post IR

**Fig. 4:**
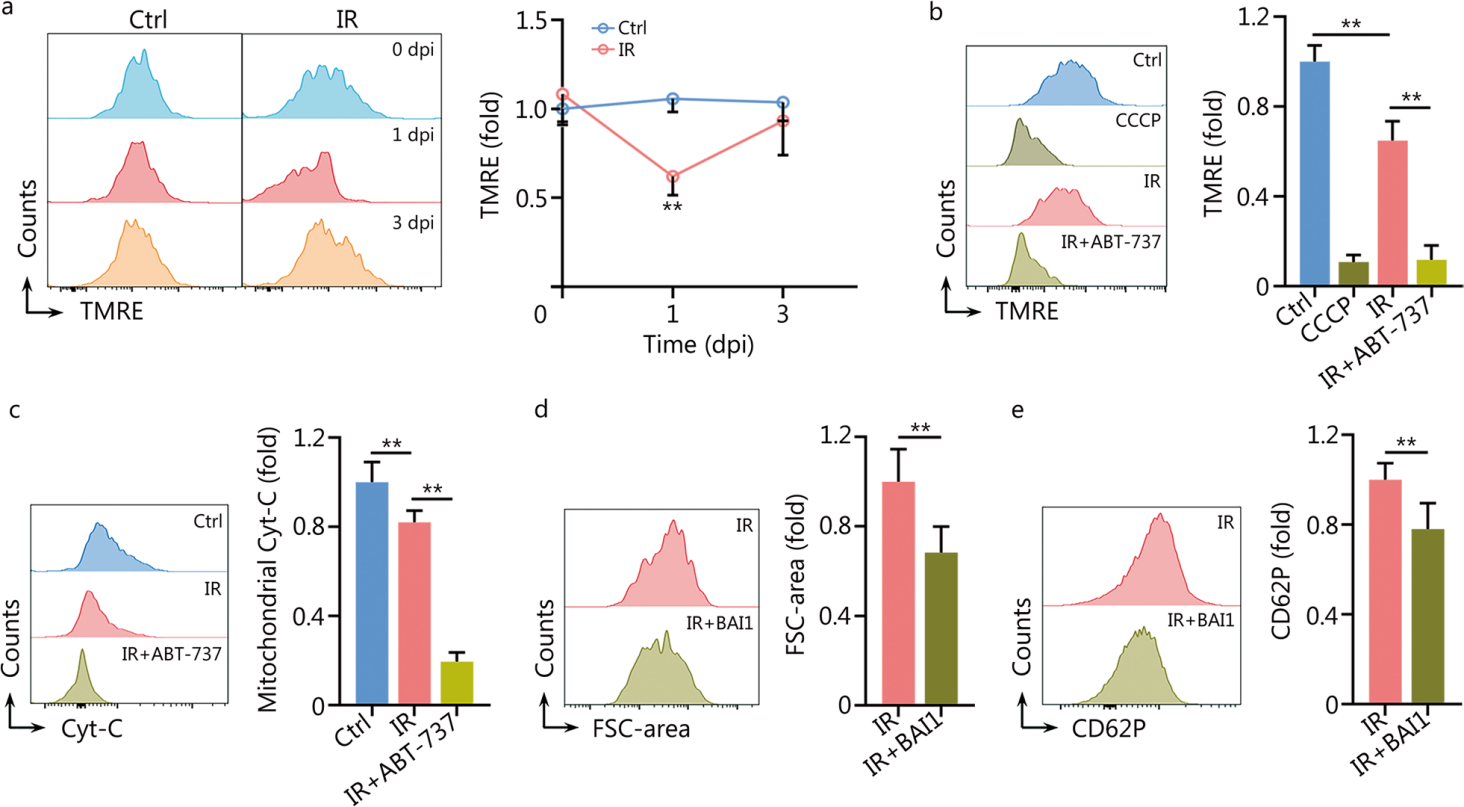
Minority MOMP is triggered in mMKs post IR. **a** Flow cytometric analysis and quantification of MMP in BM mMKs of mice at 1 and 3 dpi (*n* = 5). **b** Flow cytometric analysis and quantification of MMP in BM mMKs of mice with or without ABT-737 treatment at 1 dpi. CCCP-treated mMKs were used as the positive control for complete MOMP (*n* = 5). **c** Flow cytometric analysis and quantification of mitochondrial Cyt-C release in BM mMKs of mice with or without ABT-737 treatment at 1 dpi (*n* = 5). **d** Flow cytometric analysis and quantification of FSC-area of PLPs in Meg-01 cultures with or without BAI1 treatment at 3 dpi (*n* = 5). **e** Flow cytometric analysis and quantification of CD62P expression on PLPs from Meg-01 cultures with or without BAI1 treatment at 3 dpi (*n* = 5). Data represent mean ± standard deviation. ^**^P < 0.01, compared to Ctrl or IR as indicated. Two-tailed unpaired student’s *t*-test. MOMP mitochondrial outer membrane permeabilization, mMK mature megakaryocyte, MMP mitochondrial membrane potential, CCCP carbonyl cyanide m-chlorophenylhydrazine, Cyt-C cytochrome C, BM bone marrow, FSC forward scatter, PLP platelet-like particle, Ctrl control, IR ionizing radiation, dpi day post IR, TMRE tetramethylrhodamine ethyl ester

Surprisingly, the transcriptome of mMKs revealed robust activation of apoptosis signaling at 1 dpi (Fig. 2g), unlike the indistinguishable apoptotic outcome between mMKs of control and IR mice (Fig. 3a). We then interrogated whether mitochondrial apoptosis signaling was authentically executed in mMKs post IR. We firstly examined MMP, an indicator of MOMP, and observed that MMP of mMKs significantly declined at 1 dpi but rapidly recovered to the normal level at 3 dpi (Fig. 4a). Meanwhile, unlike carbonyl cyanide m-chlorophenylhydrazine-treated mMKs, whose MMP completely collapsed, MMP of mMKs only partially collapsed at 1 dpi (Fig. 4b). Meanwhile, mitochondrial Cyt-C was only partially released in mMKs at 1 dpi (Fig. 4c). However, ABT-737 treatment provoked a nearly-complete MMP collapse and mitochondrial Cyt-C release in mMKs at 1 dpi (Fig. 4b, c). Similar results were observed in Meg-01 cells as well (Additional file 1: Fig. S4a, b). These data demonstrate that minority MOMP, a dose where apoptosis induction was low [25], is triggered in mMKs post IR. Besides, the block of MOMP by a BAX inhibitor BAI1 significantly blunted the upregulation of platelet activation-related molecules including VLA-6, GPVI and CD62P in Meg-01 cells at 3 dpi (Additional file 1: Fig. S4c), as well as the FSC-area increase (Fig. 4d) and thrombin-induced CD62P upregulation of PLPs (Fig. 4e) at 3 dpi. Therefore, minority MOMP accounts for platelet enlargement and hyperreactivity post IR.

### Minority MOMP stimulates cGAS/STING in mMKs post IR

**Fig. 5:**
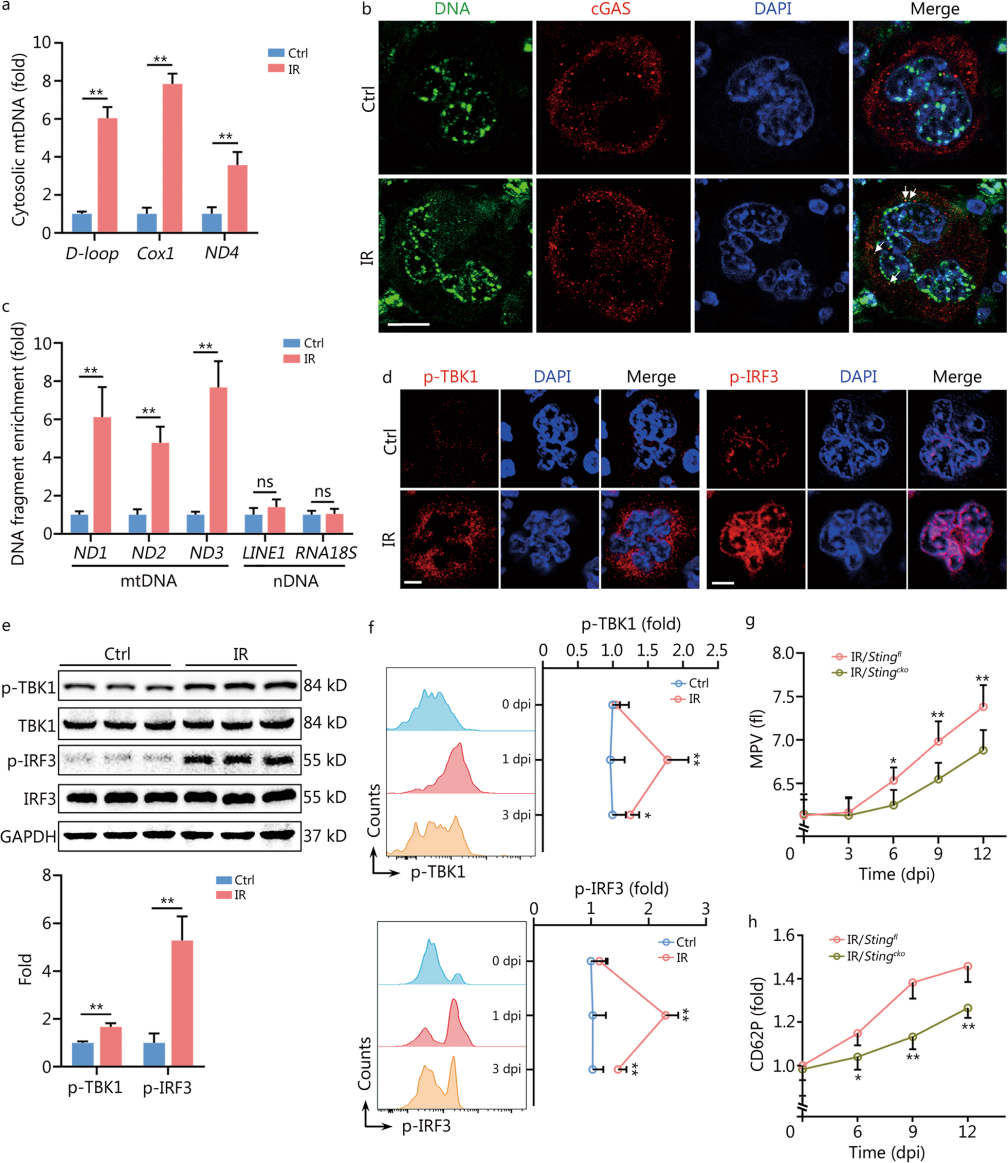
Minority MOMP stimulates cGAS/STING in mMKs post IR. **a** Relative mtDNA amounts in cytosols of mouse BM mMKs at 1 dpi were assessed by quantitative polymerase chain reaction (*n* = 3). **b** Colocalization of cytosolic DNA with cGAS in BM mMKs of mice at 1 dpi was assessed by immunofluorescence staining with antibodies specific for DNA and cGAS. Scale bar = 20 μm. The arrow indicates cGAS and DNA colocalization. **c** Relative enrichment of DNA fragments as indicated from FLAG-cGAS immunoprecipitants in Meg-01 cells at 1 dpi (*n* = 3). **d** p-TBK1 and p-IRF3 expression in BM mMKs of mice at 1 dpi were assessed by immunofluorescence staining. Scale bar = 20 μm. **e** Western blotting analysis and quantification of p-TBK1 and p-IRF3 expression in BM mMKs of mice at 1 dpi (*n* = 3). **f** Flow cytometric analysis and quantification of p-TBK1 and p-IRF3 expression in BM mMKs of mice at indicated dpi (*n* = 5). **g** MPV in peripheral blood of *Sting*^*fl*^ and *Sting*^*cko*^ mice at indicated dpi (*n* = 5). **h** CD62P expression in response to thrombin on washed platelets from *Sting*^*fl*^ and *Sting*^*cko*^ mice at indicated dpi (*n* = 4–5). Data represent mean ± standard deviation. ^*^P < 0.05, ^**^P < 0.01, compared to Ctrl or IR/*Sting*^*fl*^ as indicated. Two-tailed unpaired student’s *t*-test. ns non-significance, MOMP mitochondrial outer membrane permeabilization, mMK mature megakaryocyte, cGAS cyclic GMP-AMP synthase, STING stimulator of interferon genes, Ctrl control, IR ionizing radiation, mtDNA mitochondrial DNA, BM bone marrow, dpi day post IR, TBK1 TANK-binding kinase 1, IRF3 interferon regulatory factor 3, MPV mean platelet volume, Cox1 cytochrome c oxidase subunit 1, ND1-4 NADH dehydrogenase subunit 1–4, LINE1 long interspersed nuclear element 1, RNA18S 18S ribosomal RNA, nDNA nuclear DNA, DAPI 4′,6-diamidino-2-phenylindole, GAPDH glyceraldehyde-3-phosphate dehydrogenase

Interestingly, we noted that innate immune signaling pathways especially DNA virus infection and cytosolic DNA sensing pathways were robustly activated in mMKs at 1 dpi (Fig. 2g). As known, minority MOMP can stimulate innate immune signaling through releasing mtDNA, hence inducing cell-intrinsic adaptive responses [26]. cGAS is the primary DNA sensor in mammals, stimulation of which activates the adaptor protein STING [27]. Accordingly, the accumulation of cytosolic mtDNA (Fig. 5a) as well as the colocalization of DNA with cGAS (Fig. 5b) were dramatically increased in mMKs at 1 dpi. In vitro, colocalization of DNA with cGAS was also observed in Meg-01 cells at 1 dpi (Additional file 1: Fig. S5a) and the coprecipitated DNA fragments with cGAS showed an enrichment of mtDNA without evidence of nDNA such as the most abundant nDNA sequences, long interspersed nuclear element 1 and 18S ribosomal RNA (Fig. 5c). In accordance, TBK1 and IRF3, the key kinases downstream of the cGAS-STING pathway, were significantly activated in mMKs post IR both in vivo (Fig. 5d-f) and in vitro (Additional file 1: Fig. S5b–d). Besides, the activation of TBK1 and IRF3 was significantly quenched at 3 dpi (Fig. 5f), paralleling mitochondrial recovery (Fig. 4a). Furthermore, BAI1 treatment significantly suppressed IR-induced activation of TBK1 and IRF3 in Meg-01 cells at 1 dpi (Additional file 1: Fig. S5e). To determine the functional relevance of cGAS/STING activation, STING was specifically deleted in MKs (*Sting*^*cko*^) using Cre-loxp recombination with Pf4-cre mice and *Sting*^*fl*^ mice. Surprisingly, the MPV (Fig. 5g) and thrombin-induced CD62P upregulation on platelets (Fig. 5h) were significantly reduced in *Sting*^*cko*^ mice from 6 dpi. Similar results were observed in Meg-01 cells with cGAS/STING deficiency (Additional file 1: Fig. S5f–i). Therefore, cGAS/STING is stimulated by minority MOMP in surviving mMKs and provokes the production of large and hyperreactive platelets post IR.

### IFN-inducible GTPase GBP2 mediates the production of large and hyperreactive platelets post IR

**Fig. 6:**
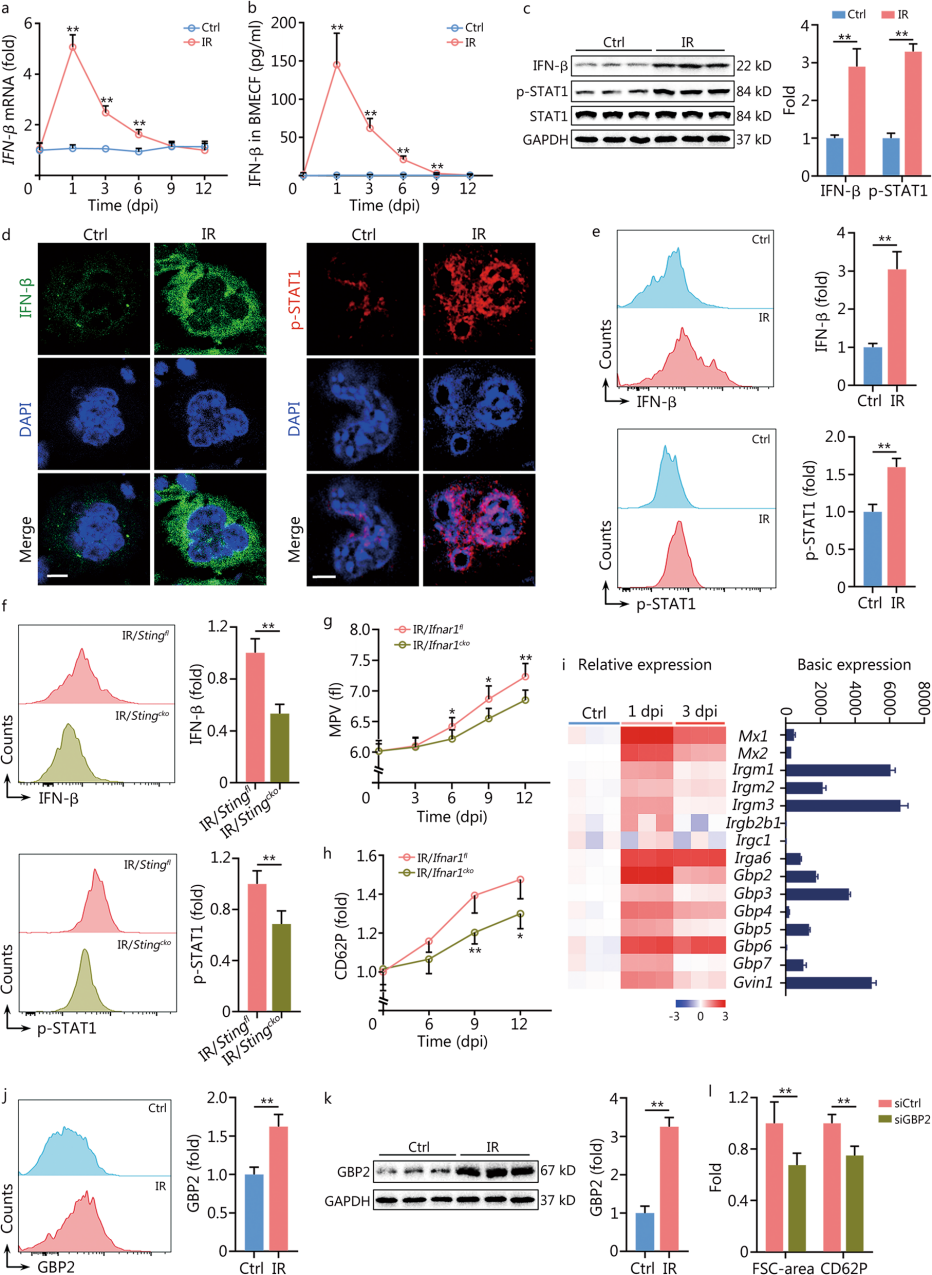
IFN-inducible GTPase GBP2 mediates production of large and hyperreactive platelets post IR. **a** Relative IFN-β mRNA expression in BM mMKs of mice at indicated dpi (*n* = 3). **b** IFN-β level in BM extracellular fluid of mice at indicated dpi was assessed by ELISA (*n* = 5). **c** Western blotting analysis and quantification of IFN-β and p-STAT1 expression in BM mMKs of mice at 1 dpi (*n* = 3). **d** IFN-β and p-STAT1 expression in BM mMKs of mice at 1 dpi were assessed by immunofluorescence staining. Scale bar = 20 μm. **e** Flow cytometric analysis and quantification of IFN-β and p-STAT1 expression in BM mMKs of mice at 1 dpi (*n* = 5). **f** Flow cytometric analysis and quantification of IFN-β and p-STAT1 expression in BM mMKs of *Sting*^*fl*^ and *Sting*^*cko*^ mice at 1 dpi (*n* = 5). **g** MPV in peripheral blood of *Ifnar1*^*fl*^ and *Ifnar1*^*cko*^ mice at indicated dpi (*n* = 5). **h** CD62P expression in response to thrombin on washed platelets from *Ifnar1*^*fl*^ and *Ifnar1*^*cko*^ mice at indicated dpi (*n* = 4–5). **i** Basic and relative mRNA expression of IFN-inducible GTPases as assessed by microarray in BM mMKs of mice at indicated dpi (*n* = 3). **j** Flow cytometric analysis and quantification of GBP2 expression in BM mMKs of mice at 3 dpi (*n* = 5). **k** Western blotting analysis and quantification of GBP2 expression in BM mMKs of mice at 3 dpi (*n* = 3). **l** FSC-area and CD62P expression in response to thrombin of PLPs from Meg-01 cultures with or without siGBP2 treatment at 3 dpi (*n* = 5). Data represent mean ± standard deviation. ^*^P < 0.05, ^**^P < 0.01, compared to Ctrl, IR/*Sting*^*fl*^, IR/*Ifnar1*^*fl*^ or siCtrl as indicated. Two-tailed unpaired student’s *t*-test. IFN interferon, GBP2 guanylate-binding protein 2, mMK mature megakaryocyte, Ctrl control, IR ionizing radiation, BM bone marrow, dpi day post IR, BMECF BM extracellular fluid, ELISA enzyme-linked immunosorbent assay, STAT1 signal transducer and activator of transcription 1, STING stimulator of interferon genes, IFNAR1 IFN‐α/β receptor 1, MPV mean platelet volume, FSC forward scatter, PLP platelet-like particle, siGBP2 siRNA of GBP2, DAPI 4′,6-diamidino-2-phenylindole, GAPDH glyceraldehyde-3-phosphate dehydrogenase

cGAS/STING activation is well known to elicit IFN-β response [27], which was robustly activated in mMKs post IR (Fig. 2f, g). Indeed, IFN-β production and secretion were rapidly and continuously enhanced in mMKs until 9 dpi (Fig. 6a–e), accompanied by significant activation of STAT1 (Fig. 6c-e), the primary downstream effector of IFN-β signaling. In contrast, megakaryocytic STING deficiency significantly inhibited IR-induced IFN-β response in mMKs (Fig. 6f). Similar results were observed in Meg-01 cells in vitro (Additional file 1: Fig. S6a–e). Interestingly, IFN-β production by mMKs even overwhelmed that by DCs and macrophages (Additional file 1: Fig. S6f), hinting that mMKs were a main source of IFN-β in the bone marrow post IR. Notably, IFN-β promoted the production of large and hyperreactive PLPs by Meg-01 cells in vitro (Additional file 1: Fig. S6g). However, the MPV (Fig. 6g) and thrombin-induced CD62P upregulation on platelets (Fig. 6h) were significantly reduced in *Ifnar1*^*cko*^ mice from 6 dpi. Similar results were observed in Meg-01 cells with IFN-β receptor 1 deficiency (Additional file 1: Fig. S6h, i), indicating an involvement of megakaryocytic IFN-I response.

Platelet formation and size rely on the coordinated rearrangement of the MK cytoskeleton, wherein GTPases play essential roles [28, 29]. As known, IFN-β-induced adaptive responses mostly depend on IFN-inducible GTPases, which include myxovirus resistance proteins, immunity-related GTPases, the GBPs, and the very large IFN-inducible GTPases [30]. Among them, we noticed that GBP2 had a relatively high basic expression and was predominantly upregulated in mMKs in vivo post IR (Fig. 6i–k). In vitro, Meg-01 cells also exhibited remarkably upregulated GBP2 expression post IR (Additional file 1: Fig. S7a, b). However, GBP2 knockdown significantly blunted the FSC-area increase and thrombin-induced CD62P upregulation of PLPs induced by IR (Fig. 6l) and IFN-β (Additional file 1: Fig. S7c, d). Thus, IFN-inducible GBP2 mediates the generation of large and hyperreactive platelets post IR.

### Autophagy restrains minority MOMP in mMKs post IR

**Fig. 7:**
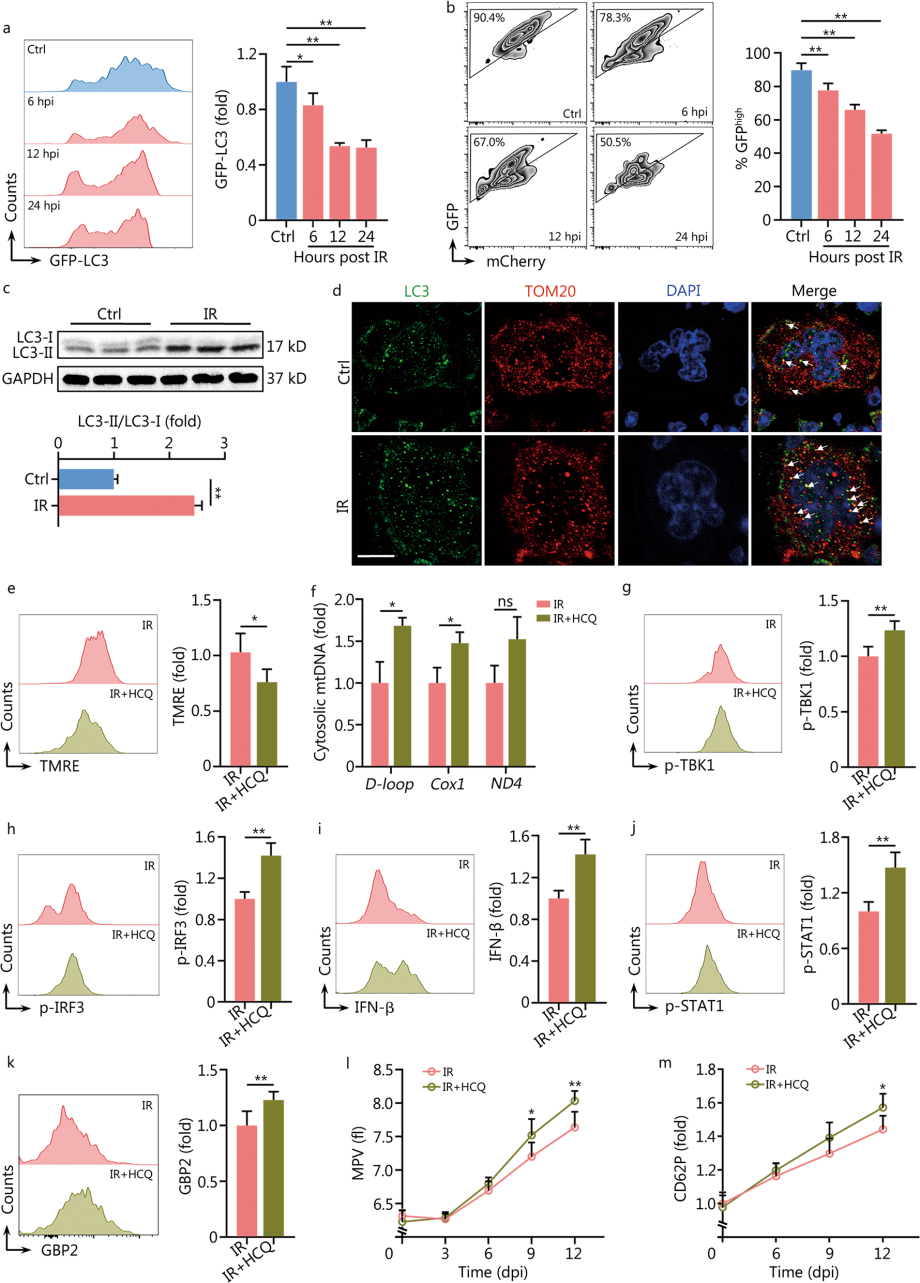
Autophagy restrains minority MOMP in mMKs post IR. **a** Flow cytometric analysis and quantification of GFP-LC3 expression in BM mMKs of GFP-LC3 mice at indicated hours post IR (hpi) (*n* = 4). **b** Flow cytometric analysis and quantification of GFP-mCherry-LC3 expression in Meg-01 cells at indicated hpi (*n* = 5). **c** Western blotting analysis and quantification of LC3 lipidation in BM mMKs of mice at 1 dpi (*n* = 3). **d** Autophagic clearance of mitochondria in BM mMKs of mice at 1 dpi was assessed by immunofluorescence staining with antibodies specific for LC3 and TOM20. Scale bar = 20 μm. **e** Flow cytometric analysis and quantification of MMP in BM mMKs of mice with or without HCQ treatment at 1 dpi (*n* = 5). **f** Relative mtDNA amounts in cytosols of BM mMKs of mice with or without HCQ treatment at 1 dpi were assessed by qPCR (*n* = 3). **g, h** Flow cytometric analysis and quantification of p-TBK1 and p-IRF3 expression in BM mMKs of mice with or without HCQ treatment at 1 dpi (*n* = 5). **i, j** Flow cytometric analysis and quantification of IFN-β and p-STAT1 expression in BM mMKs of mice with or without HCQ treatment at 1 dpi (*n* = 5). **k** Flow cytometric analysis and quantification of GBP2 expression in BM mMKs of mice with or without HCQ treatment at 3 dpi (*n* = 5). **l** MPV in peripheral blood of mice with or without HCQ treatment at indicated dpi (*n* = 6). **m** CD62P expression in response to thrombin on washed platelets from mice with or without HCQ treatment at indicated dpi (*n* = 5). Data represent mean ± standard deviation. ^*^P < 0.05, ^**^P < 0.01, compared to Ctrl or IR as indicated. Two-tailed unpaired student’s *t*-test unless stated otherwise. One-way analysis of variance (**a**, **b**). ns non-significance, MOMP mitochondrial outer membrane permeabilization, mMK mature megakaryocyte, Ctrl control, IR ionizing radiation, BM bone marrow, hpi hour post IR, dpi day post IR, GFP green fluorescent protein, LC3 microtubule-associated protein 1 light chain 3, LC3-I non-lipidized LC3, LC3-II lipidized LC3, TOM20 translocase of outer mitochondrial membrane 20, MMP mitochondrial membrane potential, HCQ hydroxychloroquine, mtDNA mitochondrial DNA, TBK1 TANK-binding kinase 1, IRF3 interferon regulatory factor 3, IFN-β interferon-β, STAT1 signal transducer and activator of transcription 1, GBP2 guanylate-binding protein 2, MPV mean platelet volume, Cox1 cytochrome c oxidase subunit 1, ND1 NADH dehydrogenase subunit 1, TMRE tetramethylrhodamine ethyl ester, DAPI 4’,6-diamidino-2-phenylindole, GAPDH glyceraldehyde-3-phosphate dehydrogenase

The self-limitation of minority MOMP indicates that mitochondrial fitness recovers quickly in surviving mMKs post IR. In mammal cells, damaged mitochondria are cleared primarily through autophagy, which is often induced by cytotoxic stimuli [31]. Using GFP-LC3 reporter mice and Meg-01 cells stably expressing GFP-mCherry-LC3, we found that autophagy was immediately invoked in mMKs post IR both in vivo (Fig. 7a) and in vitro (Fig. 7b), accompanied by dramatic LC3 lipidation (Fig. 7c, Additional file 1: Fig. S8a). Confocal immunofluorescence microscopy revealed that autophagic clearance of mitochondria was strikingly enhanced in mMKs post IR both in vivo (Fig. 7d) and in vitro (Additional file 1: Fig. S8b). Subsequently, IR mice were treated with HCQ, a potent in vivo autophagy blocker. Surprisingly, HCQ not only delayed the recovery of mitochondrial fitness (Fig. 7e) but also exacerbated the accumulation of cytosolic mtDNA (Fig. 7f) as well as the activation of cGAS/STING (Fig. 7g, h) and IFN-β response (Fig. 7i–k) in mMKs post IR. Consequently, HCQ treatment further increased the MPV (Fig. 7l) and thrombin-induced CD62P upregulation on platelets (Fig. 7m) from 9 dpi. Similar results were confirmed in vitro using Meg-01 cells with CQ treatment (Additional file 1: Fig. S8c–g). Overall, these data demonstrate that autophagy restrains minority MOMP in mMKs post IR.

## Discussion

**Fig. 8:**
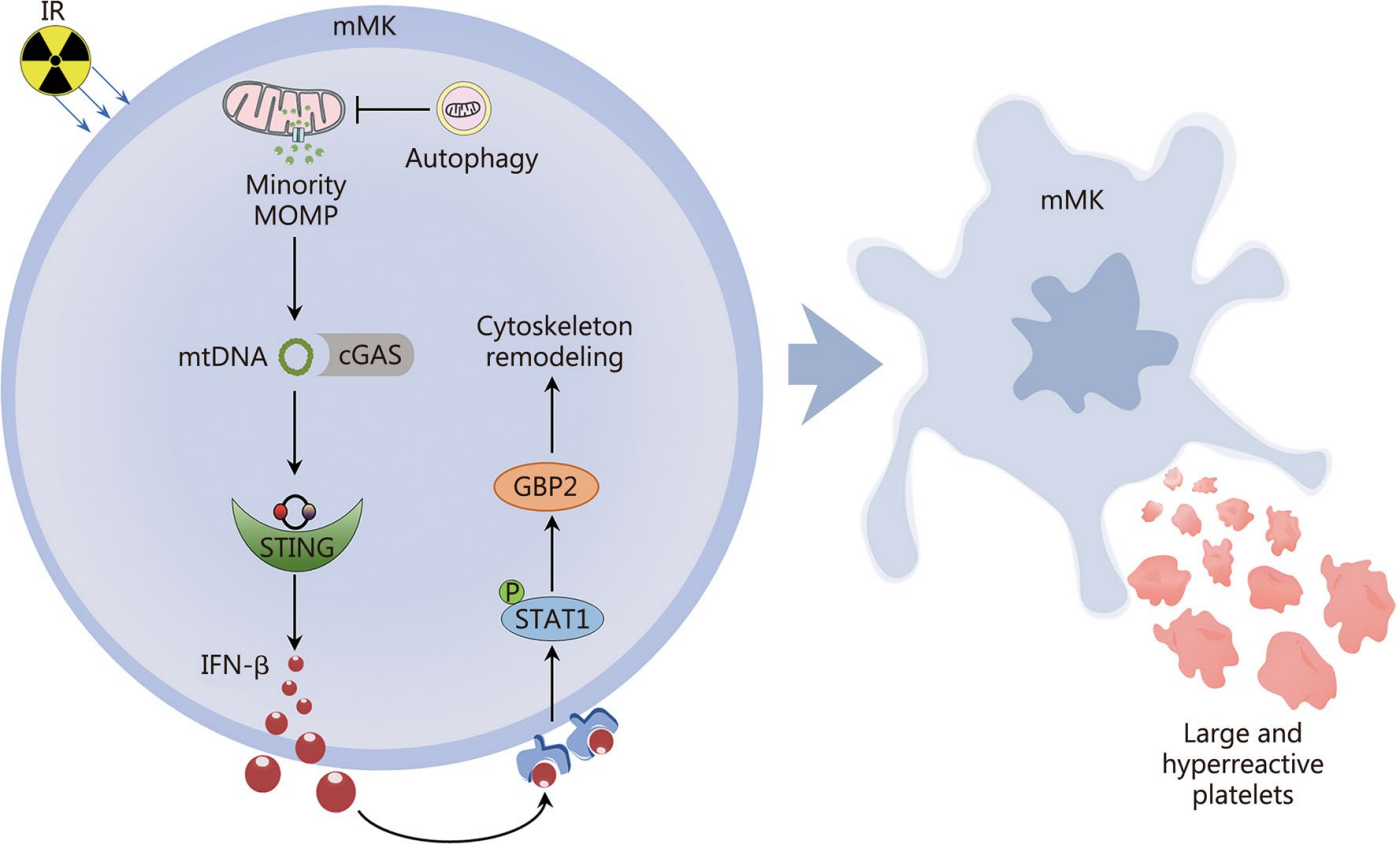
The mechanism of large and hyperreactive platelet production by megakaryocytes in response to radiation injury. IR ionizing radiation, mMK mature megakaryocyte, MOMP mitochondrial outer membrane permeabilization, mtDNA mitochondrial DNA, cGAS cyclic GMP-AMP synthase, STING stimulator of interferon genes, IFN-β interferon-β, STAT1 signal transducer and activator of transcription 1, GBP2 guanylate-binding protein 2

Although thrombopoiesis has been extensively studied, there remain significant knowledge gaps regarding how thrombopoiesis copes with the detrimental effects of thrombocytopenia, particularly the hemorrhagic tendency. Consequently, there are currently no available targeted therapies other than supportive care for thrombocytopenia. In this study, using a mouse model of radiation injury-induced thrombocytopenia, we reveal that mMKs are resistant to IR-induced apoptosis. In the face of IR, mMKs undergo minority MOMP, which stimulates the activation of the cGAS-STING pathway by releasing mtDNA. The subsequent IFN-β response then triggers the production of large and hyperreactive platelets by activating GBP2 (Fig. 8). Thus, our study uncovers a hitherto unrecognized property of mMKs, which fine-tunes the balance between platelet quantity and quality upon cytotoxicity.

Upon exposure to cytotoxic stimuli, although platelet numbers usually sharply decline, a certain number of platelets remain, while their origins are nearly undefined. Here, we show that the responses to IR are heterogeneous within the bone marrow compartment, with most hematopoietic cells including MkPs being extremely sensitive whereas mMKs being almost absolutely resistant to IR-induced apoptosis. As a result, mMKs survive IR and constitute a large portion of BMCs post IR. Meanwhile, the surviving mMKs undergo active platelet production and maintain the nadir platelet levels. All these observations are consistent with studies decades ago, in which MKs are found to be resistant to even lethal IR and to be functional in rodents for at least 7–10 d after IR [32, 33]. Mechanistically, the radiosensitivity is determined by the dynamic reprogramming of the Bcl-xL-BAX/BAK axis during MK development, with enhancement of apoptotic threshold in mMKs mediated by Bcl-xL upregulation and BAX/BAK downregulation, which could be partially explained by the TPO signaling [34].

Relative to the pathogenesis of thrombocytopenia, the functions of residual platelets during thrombocytopenia progress always get less attention. Thrombosis and hemorrhage usually co-exist in military personnel and civilians who have encountered chemical, biological, radioactive, and nuclear events [1, 2], while the underlying mechanism remains elusive. We in this study uncover that the surviving mMKs produce large and hyperreactive platelets post IR. This may serve as a first-aid strategy to compensate for the weakened hemostasis upon cytotoxicity. As known, platelet activation especially disseminated intravascular coagulation rapidly consumes circulating platelets, resulting in thrombocytopenia and bleeding tendency [9]. Based on our findings, it is conceivable that platelet hyperreactivity is implicated in the pathogenesis of thrombocytopenia and even hemorrhage, thus reconciling the contradiction as mentioned above.

Notably, although apoptosis fails, minority MOMP develops in the surviving mMKs. Subsequently, minority MOMP stimulates the cGAS-STING pathway in mMKs by releasing mtDNA into the cytosol and subsequently elicits IFN-β production and secretion. Molecularly, we show that an IFN-inducible GTPase GBP2 mediates the production of large and hyperreactive platelets by mMKs. Strikingly, we also find that the cGAS-STING pathway in mMKs is even more proficient in IFN-β production, overwhelming that in DCs and macrophages in the context of IR. Together with the recent reports that MKs possess immune characteristics [35–37] as well as the pivotal role of platelet activation in pathogen restriction and tissue injury repair [11], this study substantially extends our understanding of the cellular and molecular basis of innate immunity. On the other hand, we also reveal that mMKs have evolved self-protective strategies to restrain minority MOMP, such as autophagic removal of permeabilized mitochondria as we demonstrated in the present study, highlighting a tight regulation of megakaryocytic mitochondrial fitness and platelet homeostasis under physiopathological conditions.

Platelet size and granule cargo allocation are primarily determined by the cytoskeletal dynamics, which are mostly regulated by the GTPases [28, 29]. The regulatory roles of small GTPases in thrombopoiesis and platelet function have been extensively described by plenty of studies [29], while the actions of large GTPases in MK and platelet biology are scarcely defined. In this study, we identify that GBP2 as a large GTPase is preferentially upregulated in mMKs in response to IFN-β and that enhanced GBP2 activity promotes the production of large platelets, which allows the allocation of more granules from mMKs. Actually, although GBPs are well known to function as a major nexus of anti-infective immunity [38], their role in pathogen restriction is relatively weaker than other IFN-inducible GTPases [30]. Recently, increasing studies have spotlighted their roles in tumorigenesis, metastasis and bone turnover through remodeling cytoskeleton [38, 39]. Among GBPs, only GBP2 and GBP5 are prenylated to facilitate membrane localization, similar to small GTPases [30]. Besides, apart from STAT1, NF-κB and STAT3 which are downstream effectors of a variety of cytokines also transcriptionally upregulate GBP2 expression [38]. These lines of evidence infer a dominant role of GBP2 in dictating platelet size and reactivity in a wide range of physiopathological conditions.

IFN-I is closely associated with platelet hyperreactivity and thromboembolism in mammals [40–42]. Thrombocytopenia and thrombosis are also common in microbial infections [43], which are nearly inevitably associated with IFN-I response [27]. In addition, aging and aging-related diseases such as cardiovascular disease and metabolic disease, in which mitochondrial damage and cGAS/STING activation are implicated [19, 44], are always associated with platelet enlargement and hyperreactivity [17, 45, 46]. In the same line of evidence, diseases in which cGAS/STING is constitutively activated and/or IFN-β level is elevated, including autoimmune diseases such as systemic lupus erythematosus [41, 42], autoinflammatory diseases such as STING-associated vasculopathy with onset in infancy [47], are also associated with high MPV and/or thrombotic risk. Based on our findings, IFN-β response may play a pathogenetic role in these scenarios, while the therapeutic intervention of IFN-β response opens new opportunities to decrease thrombotic morbidity and mortality.

## Conclusions

Above all, this study substantially advances our understanding of the link of innate immunity with thrombopoiesis as well as with thrombosis and hemostasis. The findings represent an important paradigm for the understanding of thrombosis and hemostasis in physiopathological conditions associated with cytotoxicity, and have profound implications for the diagnosis and treatment of platelet disorders.

### Supplementary Information

**Additional file 1: Table S1** Antibodies used in Western blotting (WB), immunofluorescence, and flow cytometry. **Table S2** Primer sequences for mitochondrial DNA (mtDNA) and nuclear DNA (nDNA) analysis. **Table S3** siRNA sequences used for human RNA interference. **Table S4** Primer sequences for mRNA expression analysis. **Fig. S1** Number of bone marrow cells in mice at indicated dpi. **Fig. S2** Platelets are molecularly and functionally hyperreactive post IR. **Fig. S3** The inherently-high pro-survival threshold confers radioresistance onto mMKs. **Fig. S4** Minority MOMP is triggered in Meg-01 post IR. **Fig. S5** Minority MOMP stimulates cGAS/STING in Meg-01 post IR. **Fig. S6** IFN-β response is triggered in mMKs post IR. **Fig. S7** IFN-inducible gene GBP2 mediates production of large and hyperreactive platelets post IR. **Fig. S8** Autophagy restrains minority MOMP in Meg-01 post IR.

## Data Availability

All data generated or analyzed during this study are included in this published article and its supplementary information files. The RNA-seq datasets analyzed during the current study are available in the NCBI Gene Expression Omnibus under accession Number GSE222512.

## Abbreviations

ACD: Acid-citrate dextrose
BMC: Bone marrow cell
cGAS: Cyclic GMP-AMP synthase
CQ: Chloroquine
Cyt-C: Cytochrome C
DC: Dendritic cell
dpi: Day post IR
EDTA: Ethylenediamine tetraacetic acid
FSC: Forward scatter
GBP2: Guanylate-binding protein 2
HCQ: Hydroxychloroquine
IFN-β: Interferon-β
IR: Ionizing radiation
IRF3: Interferon regulatory factor 3
MK: Megakaryocyte
MkP: MK progenitor
mMK: Mature MK
MMP: Mitochondrial membrane potential
MOMP: Mitochondrial outer membrane permeabilization
MPV: Mean platelet volume
mtDNA: Mitochondrial DNA
nDNA: Nuclear DNA
PBS: Phosphate‐buffered saline
PLP: Platelet-like particle
PMA: Platelet-monocyte aggregate
PMP: Platelet-derived microparticle
qPCR: Quantitative polymerase chain reaction
STAT1: Signal transducer and activator of transcription 1
STING: Stimulator of IFN genes
TBK1: TANK-binding kinase 1
TPO: Thrombopoietin
