# Supplementary material for: Apoptosis-resistant megakaryocytes produce large and hyperreactive platelets in response to radiation injury
Source: Mil Med Res. 2023 Dec 19;10:66. doi: 10.1186/s40779-023-00499-z (PMC10729570; doi:10.1186/s40779-023-00499-z)
Supplement: Supplementary file 1 — Additional file 1: Table S1 Antibodies used in Western blotting (WB), immunofluorescence, and flow cytometry. Table S2 Primer sequences for mitochondrial DNA (mtDNA) and nuclear DNA (nDNA) analysis. Table S3 siRNA sequences used for human RNA interference. Table S4 Primer sequences for mRNA expression analysis. Fig. S1 Number of bone marrow cells in mice at indicated dpi. Fig. S2 Platelets are molecularly and functionally hyperreactive post IR. Fig. S3 The inherently-high pro-survival threshold confers radioresistance onto mMKs. Fig. S4 Minority MOMP is triggered in Meg-01 post IR. Fig. S5 Minority MOMP stimulates cGAS/STING in Meg-01 post IR. Fig. S6 IFN-β response is triggered in mMKs post IR. Fig. S7 IFN-inducible gene GBP2 mediates production of large and hyperreactive platelets post IR. Fig. S8 Autophagy restrains minority MOMP in Meg-01 post IR. [file 40779_2023_499_MOESM1_ESM.pdf]

Table S1 Antibodies used in Western blotting (WB), immunofluorescence, and flow cytometry

Antibody	Origin	Catalog number	Assay
CD41a monoclonal antibody (eBioMWReg30 (MWReg30)), APC	eBioscience	Cat# 17-0411-82	FC
CD42d monoclonal antibody (1C2), APC	eBioscience	Cat# 17-0421-82	FC
CD42d monoclonal antibody (1C2), PE	eBioscience	Cat# 12-0421-82	FC
CD41a monoclonal antibody [eBioMWReg30 (MWReg30)], FITC	eBioscience	Cat# 11-0411-85	FC
CD41a monoclonal antibody [eBioMWReg30 (MWReg30)], Biotin	eBioscience	Cat# 13-0411-82	FC
PE anti-mouse/rat CD62P (P-selectin) antibody	Biolegend	Cat# 148306	FC
Integrin alpha IIb beta 3 (GPIIb/IIIa) activation, PE	Emfret Analytics	Cat# M023-2	FC
Biotin mouse hematopoietic lineage cocktail	eBioscience	Cat# 88-7774-75	FC
Brilliant Violet 510™ Streptavidin	Biolegend	Cat# 405234	FC
Ly-6A/E (Sca-1) monoclonal antibody (D7), PerCP-Cyanine5.5	eBioscience	Cat# 45-5981-82	FC
CD117 (c-Kit) monoclonal antibody (2B8), APC-eFluor™ 780	eBioscience	Cat# 47-1171-82	FC
CD150 monoclonal antibody (mShad150), PE-Cyanine7	eBioscience	Cat# 25-1502-82	FC
CD45 monoclonal antibody (HI30), eFluor 450	eBioscience	Cat# 48-0459-42	FC
CD115 (c-fms) monoclonal antibody (AFS98), PE-Cyanine7	eBioscience	Cat# 25-1152-80	FC
CD11b monoclonal antibody (M1/70), APC	eBioscience	Cat# 17-0112-82	FC
Ly-6G/Ly-6C (Gr-1) monoclonal antibody (RB6-8C5), PE	eBioscience	Cat# 12-5931-82	FC
APC anti-mouse CD80	eBioscience	Cat# 17-0801-82	FC
PerCP-Cyanine5.5 anti-mouse CD11c	eBioscience	Cat# 45-0114-82	FC
Anti-human CD41 antibody, APC	Biolegend	Cat# 303710	FC
Anti-human CD62P (P-Selectin) antibody, PE	Biolegend	Cat# 304906	FC

Antibody	Origin	Catalog number	Assay
Annexin V- eFluor 450 apoptosis detection kit	eBioscience	Cat# 88-8006-74	FC
Bcl-xL monoclonal antibody (C.85.1)	Thermo Fisher Scientific	Cat# MA5-15142	FC
MCL1 recombinant rabbit monoclonal antibody (SI16-04)	Thermo Fisher Scientific	Cat# MA5-32060	FC
BAX monoclonal antibody (6A7)	Thermo Fisher Scientific	Cat# MA5-14003	FC
BAK1 monoclonal antibody (4C2)	Thermo Fisher Scientific	Cat# MA5-36225	FC
Cleaved caspase-3 (Asp175) (D3E9) rabbit mAb (Alexa Fluor® 647 Conjugate)	Cell Signaling Technology	Cat# 9602S	FC
Alexa Fluor® 647 anti-cytochrome c antibody	Biolegend	Cat# 612310	FC
TBK1 antibody	Abcam	Cat# ab40676	WB
p-TBK1 (Ser172) antibody	Cell Signaling Technology	Cat# 5483	WB, IF
p-IRF3 (Ser396) antibody	Cell Signaling Technology	Cat# 29047	WB, IF
IRF3 antibody	Cell Signaling Technology	Cat# 4302	WB
p-TBK1 (Ser172) antibody (Alexa Fluor 647 Conjugate)	Cell Signaling Technology	Cat# 14590	FC
p-IRF3 (Ser396) antibody (Alexa Fluor 647 Conjugate)	Cell Signaling Technology	Cat# 10327	FC
IFN-β antibody	Abcam	Cat# ab85803	WB, IF, FC
Anti-IFN-β (EPR22186-266)	Abcam	Cat# ab218229	WB
IFN-β antibody (MIB-8C4.1)	Santa Cruz Biotechnology	Cat# sc-53586	FC, IF
p-STAT1 (Tyr701) antibody	Cell Signaling Technology	Cat# 9167	WB, IF
STAT1 antibody	Cell Signaling Technology	Cat# 14994	WB
p-STAT1 (Ser727) antibody (Alexa Fluor 647 Conjugate)	Biolegend	Cat# 686412	FC
Anti-GBP2 antibody	Abcam	Cat# ab203238	WB
GBP2 polyclonal antibody	Thermo Fisher Scientific	Cat# PA5-112426	FC

Antibody	Origin	Catalog number	Assay
GAPDH (D16H11) XP® Rabbit mAb	Cell Signaling Technology	Cat# 5174S	WB
cGAS antibody	Abcam	Cat# ab224144	IF
cGAS antibody	Santa Cruz Biotechnology	Cat# sc-515777	IF
Tom20 antibody	Santa Cruz Biotechnology	Cat# sc-17764	IF
LC3B antibody	Novus Biologicals	Cat# NB100-2220	WB, IF
DNA antibody	Millipore	Cat# CBL186	IF
Monoclonal ANTI-Flag® M2 antibody	Sigma-Aldrich	Cat# F1804	ChIP
Goat anti-mouse IgG (H + L) cross-adsorbed secondary antibody, Alexa Fluor 488	Thermo Fisher Scientific	Cat# A-11001	IF
Goat anti-mouse IgG (H + L) cross-adsorbed secondary antibody, Alexa Fluor 546	Thermo Fisher Scientific	Cat# A-11003	IF
Goat anti-rabbit IgG (H + L) cross-adsorbed secondary antibody, Alexa Fluor 488	Thermo Fisher Scientific	Cat# A-11008	IF
Goat anti-rabbit IgG (H + L) cross-adsorbed secondary antibody, Alexa Fluor 546	Thermo Fisher Scientific	Cat# A-11010	IF
Goat anti-rabbit IgG (H + L) cross-adsorbed secondary antibody, Alexa Fluor 647	Thermo Fisher Scientific	Cat# A-21244	FC
Goat anti-mouse IgG (H + L) cross-adsorbed secondary Antibody, Alexa Fluor 647	Thermo Fisher Scientific	Cat# A-21235	FC
HRP labeled goat anti-rabbit IgG (H + L)	Abcam	Cat# 205718	WB
HRP labeled goat anti-Mouse IgG (H + L)	Abcam	Cat# 205719	WB

IF immunofluorescence, *FC* flow cytometry, *ChIP* chromatin immunoprecipitation, *LC3B* microtubule-associated protein 1 light chain 3B, *TOM20* translocase of outer mitochondrial membrane 20, *cGAS* cyclic GMP-AMP synthase, *STING* stimulator of interferon genes, *TBK1* TANK-binding kinase 1, *IRF3* interferon regulatory factor 3, *IFN-β* interferon-β, *STAT1* signal transducer and activator of transcription 1, *GBP2* guanylate-binding protein 2, *GAPDH* glyceraldehyde-3-phosphate dehydrogenase, *FITC* fluorescein isothiocyanate, *PE* phycoerythrin, *APC* allophycocyanin, *PerCP* peridinin chlorophyll protein, *IgG* Allophycocyanin, *HRP* horseradish peroxidase

Table S2 Primer sequences for mitochondrial DNA (mtDNA) and nuclear DNA (nDNA) analysis

**Origin**	**Gene**	**Forward primer (5' - 3')**	**Reverse primer (5' - 3')**
mtDNA (mouse)	*D-loop*	AATCTACCATCCTCCGTGAAACC	TCAGTTTAGCTACCCCCAAGTTTAA
	Cox1	GCCCCAGATATAGCATTCCC	GTTCATCCTGTTCCTGCTCC
	ND4	AACGGATCCACAGCCGTA	AGTCCTCGGGCCATGATT
mtDNA (human)	*ND1*	CCCTAAAACCCGCCACATCT	GAGCGATGGTGAGAGCTAAGGT
	ND2	ACCATCTTTGCAGGCACACT	GCTTCTGTGGAACGAGGGTT
	ND4	TTCCTCCGACCCCCTAACAA	GATAAGTGGCGTTGGCTTGC
nDNA (human)	*LINE1*	AGAACGCCACAAAGATACTCCTCG	CTCTCTTCTGGCTTGTAGGGTTTCTG
	RNA18S	TGCCCTATCAACTTTCGATGGTAGTC	TTGGATGTGGTAGCCGTTTCTCA

Table S3 siRNA sequences used for human RNA interference

**Gene**	**Sense**	**Antisense**
cGAS	CCAACACUCGUGCAUAUUATT	UAAUAUGCACGAGUGUUGGTT
STING	GCAUCAAGGAUCGGGUUUATT	UAAACCCGAUCCUUGAUGCTT
IFNAR1	CCUACUUCCUCCAGUCUUUTT	AAAGACUGGAGGAAGUAGGTT
GBP2	GCCCUUUAGAAGAAGAUGUTT	ACAUCUUCUUCUAAAGGGCTT
Negative control	UCCUCCGAACGUGUCACGUTT	ACGUGACACGUUCGGAGAATT

Table S4 Primer sequences for mRNA expression analysis

**Source**	**Gene**	**Forward primer (5' - 3')**	**Reverse primer (5' - 3')**
Human	*Bcl-xL*	AAAAGATCTTCCGGGGGCTG	TCCACAAAAGTATCCTGTTCAAAGC
	MCL	CGACTTTTGGCCACCGGC	TAGCCAGTCCCGTTTTGTCC
	BAX	TCATGGGCTGGACATTGGAC	GCGTCCCAAAGTAGGAGAGG
	BAK	GGACCCATGCTGGAGTAAGAATAA	TTCCTGCTGATGGCGGTAAA
	VLA-6	GGCGGTGTTATGTCCTGAGTC	AATCGCCCATCACAAAAGCTC
	GPVI	TCCCGGCCATGAAGAGAAGT	TTACGTCCCCTCCTGACGAC
	CD62P	ACTGCCAGAATCGCTACACAG	CACCCATGTCCATGTCTTATTGT
	cGAS	ACATGGCGGCTATCCTTCTCT	GGGTTCTGGGTACATACGTGAAA
	STING	CCAGAGCACACTCTCCGGTA	CGCATTTGGGAGGGAGTAGTA
	IFNAR1	AACAGGAGCGATGAGTCTGTC	TGCGAAATGGTGTAAATGAGTCA
	GBP2	ATTGTGGGCCTCTATCGCAC	CCAGGTGAGGAGTTTGCCTT
	GAPDH	AGCCTCAAGATCATCAGCAA	GTCATGAGTCCTTCCACGATAC
Mouse	*IFN-β*	TGGGTGGAATGAGACTATTGTTG	CTCCCACGTCAATCTTTCCTC
	Bcl-xL	GACAAGGAGATGCAGGTATTGG	TCCCGTAGAGATCCACAAAAGT
	MCL	AAAGGCGGCTGCATAAGTC	TGGCGGTATAGGTCGTCCTC
	BAX	CCGGCGAATTGGAGATGAACT	CCAGCCCATGATGGTTCTGAT
	BAK	GTGACCTGCTTTTTGGCTGAT	GGTCTCTACGCAAATTCAGGG
	GAPDH	AGGTCGGTGTGAACGGATTTG	TGTAGACCATGTAGTTGAGGTCA

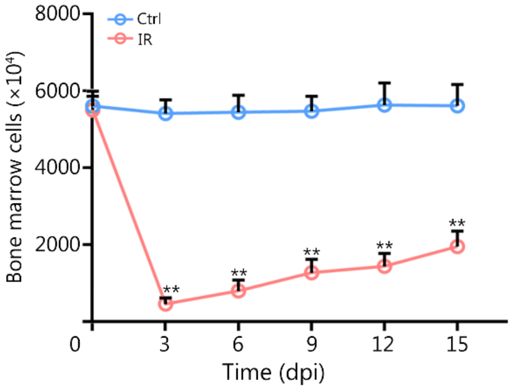

Fig. S1 Number of bone marrow cells in mice at indicated dpi. $n = 6$. Data represent mean ± standard deviation. $^{**}P < 0.01$, compared to Ctrl. Two-tailed unpaired student's *t*-test. Ctrl control, IR ionizing radiation, dpi day post IR

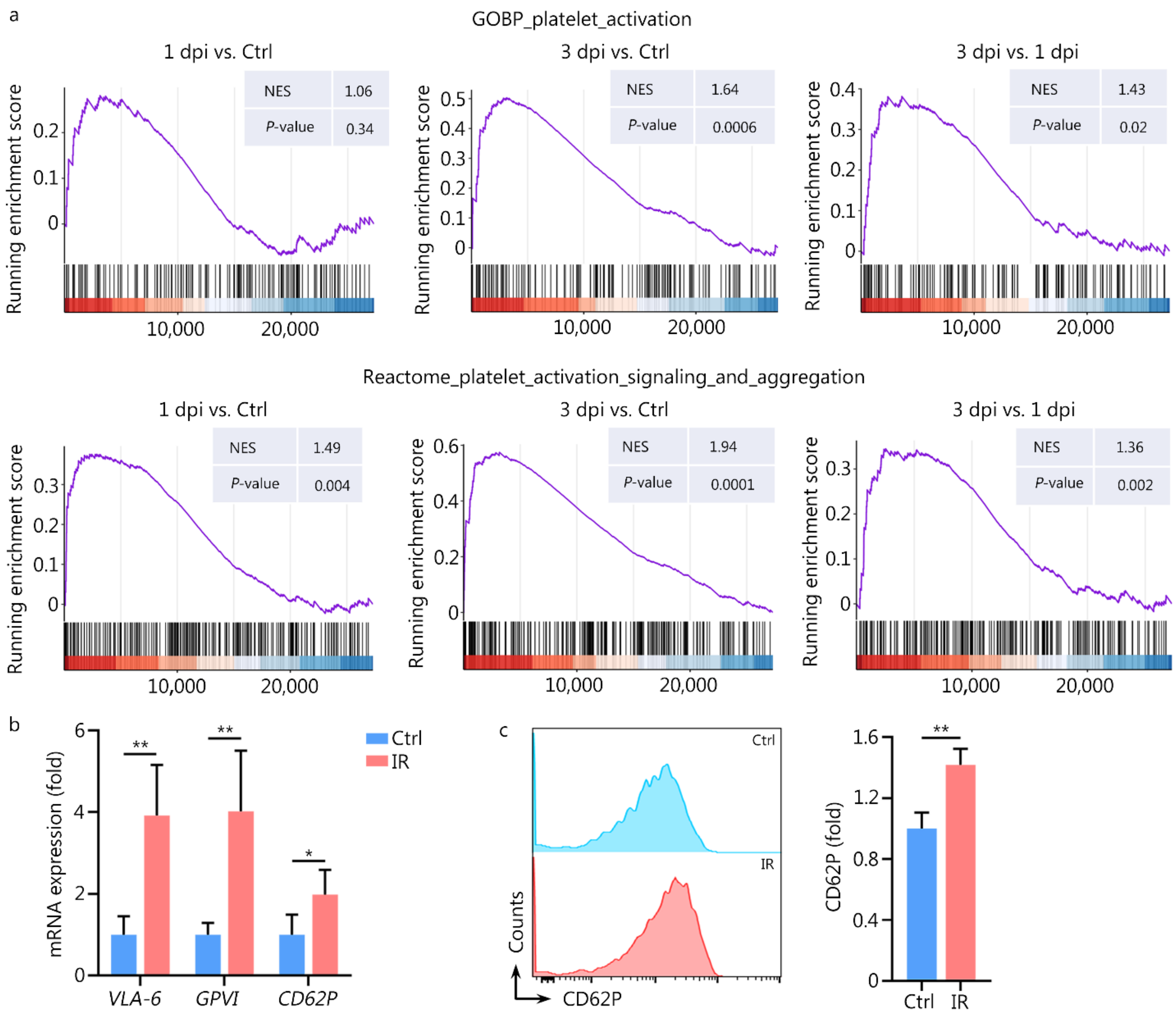

Fig. S2 Platelets are molecularly and functionally hyperreactive post IR. **a** Gene set enrichment analysis of indicated gene sets in BM mMKs of mice at 1 and 3 dpi. **b** Relative mRNA expression of *VLA-6*, *GPVI*, and *CD62P* in Meg-01 cells at 3 dpi ($n = 4$). **c** Flow cytometric analysis and quantification of CD62P expression in response to thrombin on PLPs from Meg-01 cultures at 3 dpi ($n = 5$). Data represent mean ± standard deviation. $^{*}P < 0.05$, $^{**}P < 0.01$, compared to Ctrl. Two-tailed unpaired student's t-test. mMK mature megakaryocyte, BM bone marrow, Ctrl control, IR ionizing radiation, dpi day post IR, VLA-6 integrin α6β1, GPVI glycoprotein VI, PLP platelet-like particle, NES normalized enrichment score, GOBP gene ontology-biological process

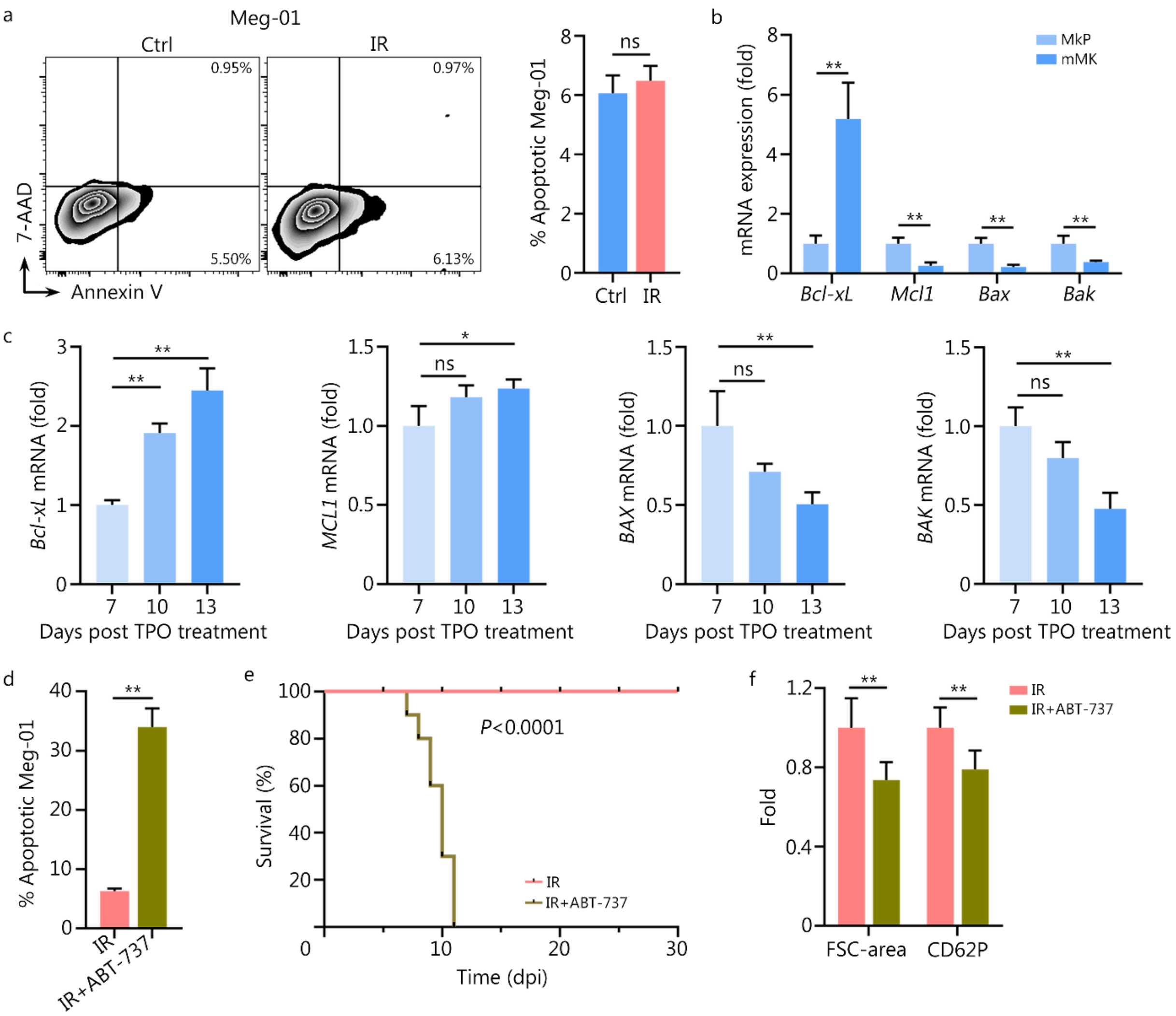

Fig. S3 The inherently high pro-survival threshold confers radioresistance onto mMKs. **a** Flow cytometric analysis and quantification of Meg-01 apoptosis at 1 dpi (*n* = 5). b Relative mRNA expression of pro-survival (*Bcl-xL* and *Mcl1*) and pro-apoptotic (*Bax* and *Bak*) Bcl-2 family in BM MkPs and mMKs of mice (*n* = 4). c Relative mRNA expression of pro-survival (*Bcl-xL* and *MCL1*) and pro-apoptotic (*BAX* and *BAK*) Bcl-2 family in human primary MKs during TPO-induced maturation (*n* = 3). **d** Flow cytometric quantification of Meg-01 apoptosis with or without ABT-737 treatment in vitro at 1 dpi (*n* = 5). **e** Survival analysis of mice with or without ABT-737 treatment post IR (*n* = 10). **f** Flow cytometric quantification of FSC-area and CD62P expression in response to thrombin of PLPs from Meg-01 cultures at 3 dpi (*n* = 5). Data represent mean ± standard deviation. ns, non-significance. $^{*}P < 0.05$, $^{**}P < 0.01$, compared to MkP, Day 7, or IR. Two-tailed unpaired student's *t*-test unless stated otherwise. One-way analysis of variance (**b**). Log-rank test (**e**). MK megakaryocyte, mMK mature MK, MkP MK progenitor, TPO thrombopoietin, Ctrl control, IR ionizing radiation, dpi day post IR, 7-AAD 7-amino-actinomycin D, PLP platelet-like particle, FSC forward scatter

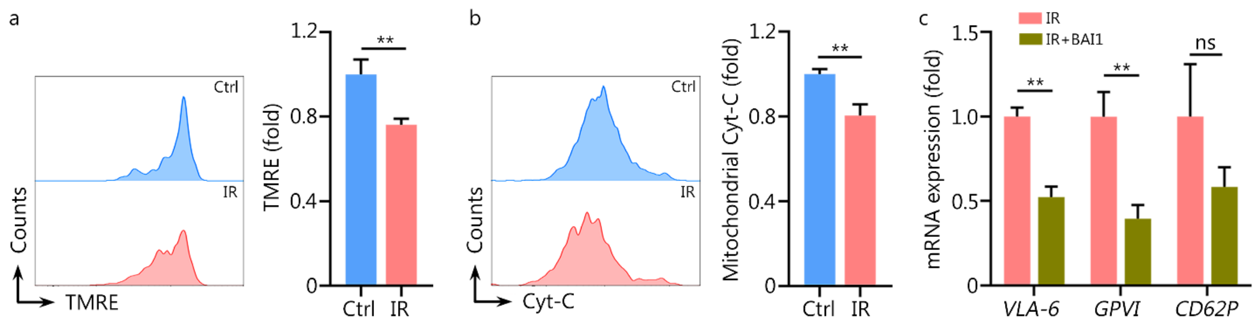

Fig. S4 Minority MOMP is triggered in Meg-01 post IR. **a** Flow cytometric analysis and quantification of MMP in Meg-01 cells at 1 dpi (n = 5). **b** Flow cytometric analysis and quantification of mitochondrial Cyt-C release in Meg-01 cells at 1 dpi. **c** Relative mRNA expression of *VLA-6*, *GPVI*, and *CD62P* in Meg-01 cells with or without BAI1 treatment at 3 dpi (n = 3). Data represent mean ± standard deviation. $^{**}P < 0.01$, compared to Ctrl or IR. Two-tailed unpaired student's t-test. ns non-significance, Ctrl control, IR ionizing radiation, dpi day post IR, MOMP mitochondrial outer membrane permeabilization, MMP mitochondrial membrane potential, Cyt-C cytochrome C, TMRE tetramethylrhodamine ethyl ester, VLA-6 integrin α6β1, GPVI glycoprotein VI

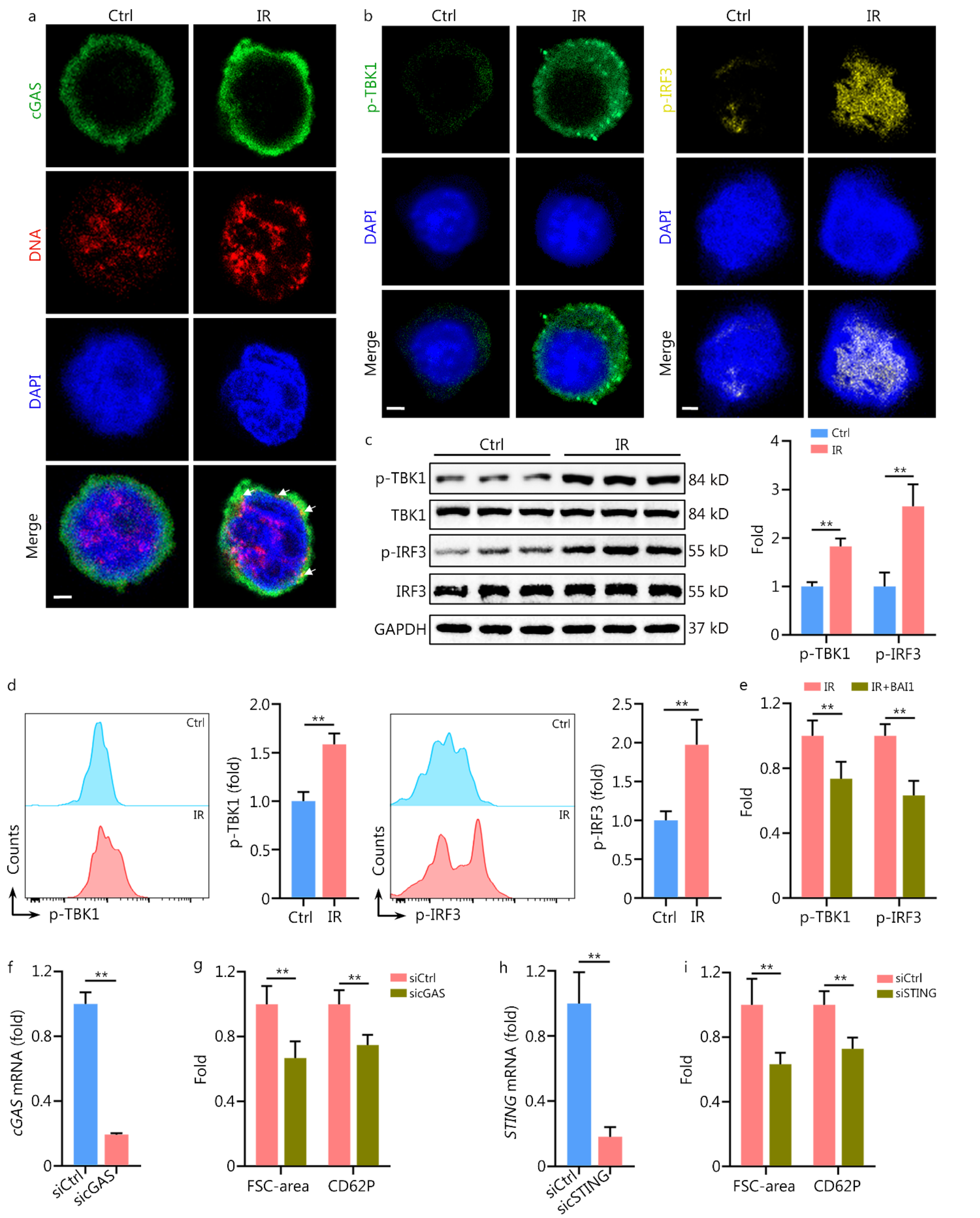

Fig. S5 Minority MOMP stimulates cGAS/STING in Meg-01 post IR. **a** Colocalization of cytosolic DNA with cGAS in Meg-01 cells at 1 dpi was assessed by immunofluorescence staining with antibodies specific for DNA and cGAS. Scale bar = 20 μm. The arrow indicates cGAS and DNA colocalization. **b** p-TBK1 and p-IRF3 expression in Meg-01 cells at 1 dpi was assessed by immunofluorescence staining. Scale bar = 20 μm. **c** Western blotting analysis and quantification of p-TBK1 and p-IRF3 expression in Meg-01 cells at 1 dpi (n = 3). d Flow cytometric analysis and

quantification of p-TBK1 and p-IRF3 expression in Meg-01 cells at 1 dpi (*n* = 5). **e** Flow cytometric quantification of p-TBK1 and p-IRF3 expression in Meg-01 cells with or without BAI1 treatment at 1 dpi (*n* = 5). **f** Relative *cGAS* mRNA expression in Meg-01 cells with or without sicGAS treatment (*n* = 3). **g** Flow cytometric quantification of FSC-area and CD62P expression in response to thrombin of PLPs from Meg-01 cultures with or without sicGAS treatment at 3 dpi (*n* = 5). **h** Relative *STING* mRNA expression in Meg-01 cells with or without siSTING treatment (*n* = 3). **i** Flow cytometric quantification of FSC-area and CD62P expression in response to thrombin of PLPs from Meg-01 cultures with or without siSTING treatment at 3 dpi (*n* = 5). Data represent mean ± standard deviation. $^{**}P < 0.01$, compared to Ctrl, IR or siCtrl. Two-tailed unpaired student's *t*-test. MOMP mitochondrial outer membrane permeabilization, cGAS cyclic GMP-AMP synthase, STING stimulator of interferon genes, Ctrl control, IR ionizing radiation, dpi day post IR, TBK1 TANK-binding kinase 1, IRF3 interferon regulatory factor 3, PLP platelet-like particle, FSC forward scatter, DAPI 4',6-diamidino-2-phenylindole, GAPDH glyceraldehyde-3-phosphate dehydrogenase, sicGAS siRNA of cGAS, siSTING siRNA of STING

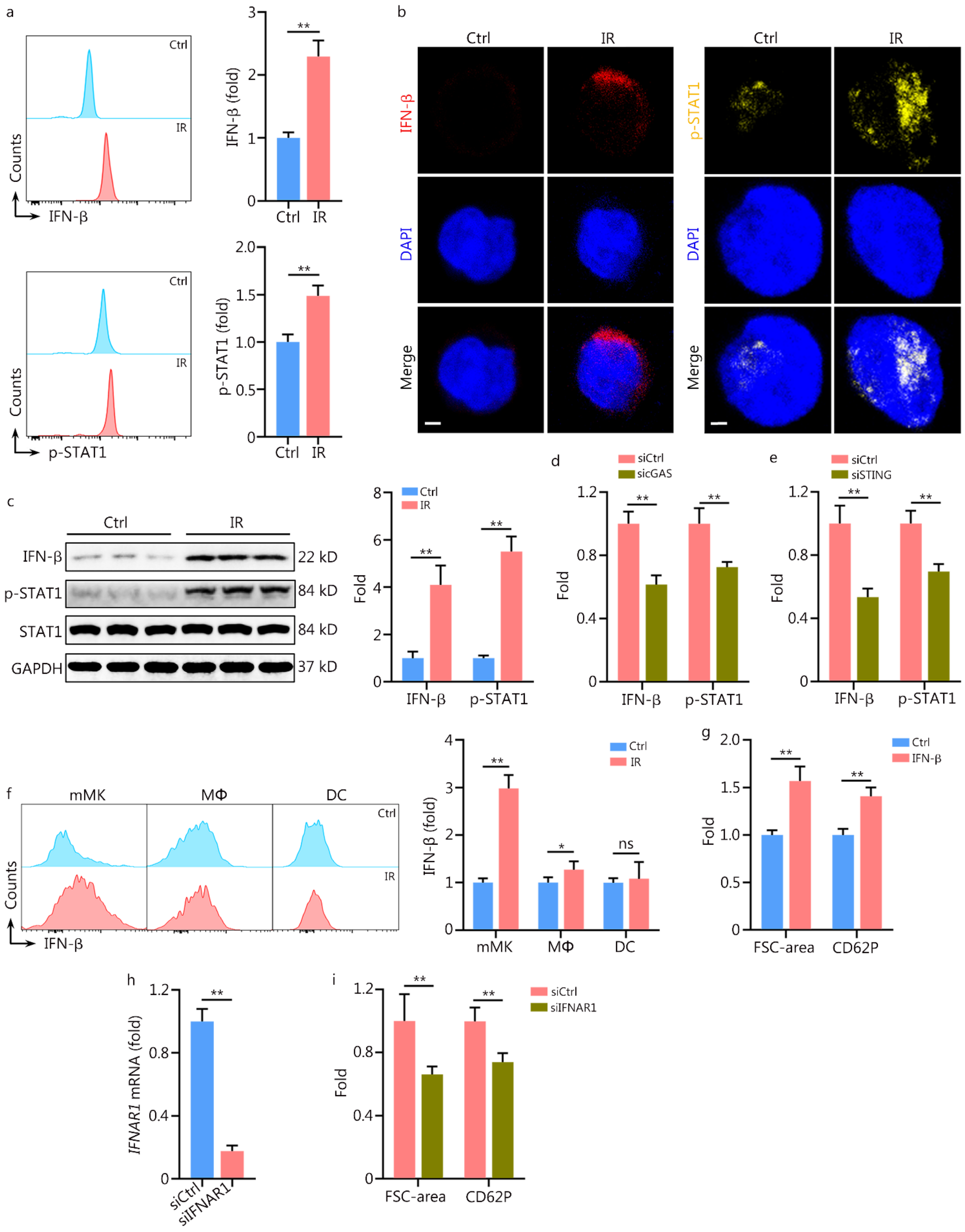

Fig. S6 IFN-β response is triggered in mMKs post IR. **a** Flow cytometric analysis and quantification of IFN-β and p-STAT1 expression in Meg-01 cells at 1 dpi (n = 5). **b** IFN-β and p-STAT1 expression in Meg-01 cells at 1 dpi was assessed by immunofluorescence staining. Scale bar = 20 μm. **c** Western blotting analysis and quantification of IFN-β and p-STAT1 expression in Meg-01 cells at 1 dpi (n = 3). **d** Flow cytometric quantification of IFN-β and p-STAT1

expression in Meg-01 cells with or without sicGAS treatment at 1 dpi (*n* = 5). **e** Flow cytometric quantification of IFN-β and p-STAT1 expression in Meg-01 cells with or without siSTING treatment at 1 dpi (*n* = 5). **f** Flow cytometric analysis and quantification of IFN-β expression in mMK, MΦ and dendritic cell DC at 1 dpi (*n* = 5). **g** Flow cytometric quantification of FSC-area and CD62P expression in response to thrombin of PLPs from Meg-01 cultures with or without IFN-β treatment at 3 dpi (*n* = 5). **h** Relative *IFNAR1* mRNA expression in Meg-01 cells with or without siIFNAR1 treatment (*n* = 3). **i** Flow cytometric quantification of FSC-area and CD62P expression in response to thrombin of PLPs from Meg-01 cultures with or without siIFNAR1 treatment at 3 dpi (*n* = 5). Data represent mean ± standard deviation. $^{*}P < 0.05$, $^{**}P < 0.01$, compared to Ctrl or siCtrl. Two-tailed unpaired student's *t*-test. ns non-significance, IFN-β interferon-β, Ctrl control, IR ionizing radiation, dpi day post IR, STAT1 signal transducer and activator of transcription 1, cGAS cyclic GMP-AMP synthase, STING stimulator of interferon genes, sicGAS siRNA of cGAS, siSTING siRNA of STING, IFNAR1 IFN-α/β receptor 1, siIFNAR1 siRNA of IFNAR1, FSC forward scatter, PLP platelet-like particle, DAPI 4',6-diamidino-2-phenylindole, GAPDH glyceraldehyde-3-phosphate dehydrogenase, MΦ macrophage, DC dendritic cell

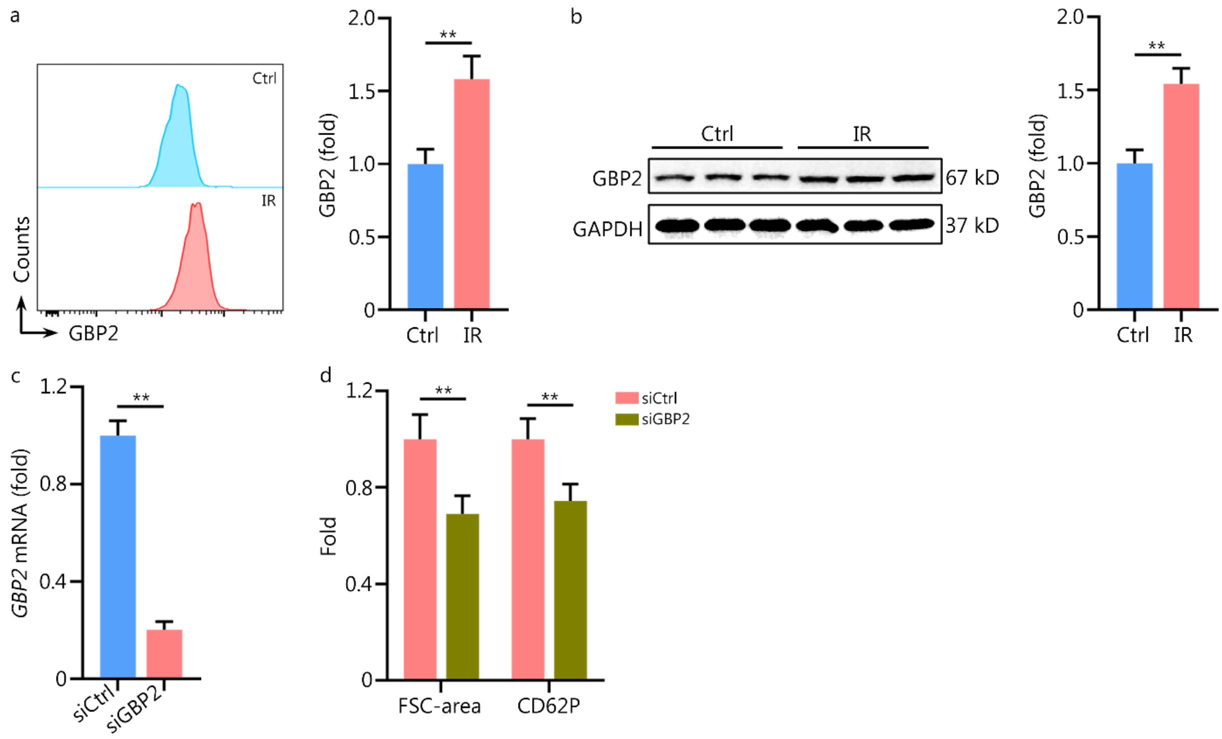

Fig. S7 IFN-inducible gene GBP2 mediates production of large and hyperreactive platelets post IR. **a** Flow cytometric analysis and quantification of GBP2 expression in Meg-01 cells at 3 dpi (n = 5). **b** Western blotting analysis and quantification of GBP2 expression in Meg-01 cells at 3 dpi (n = 3). **c** Relative *GBP2* mRNA expression in Meg-01 cells with or without siGBP2 treatment (n = 3). **d** Flow cytometric quantification of FSC-area and CD62P expression in response to thrombin of PLPs from Meg-01 cultures with or without siGBP2 treatment in the presence of IFN-β (n = 5). Data represent mean ± standard deviation. $^{*}P < 0.05$, $^{**}P < 0.01$, compared to Ctrl or siCtrl. Two-tailed unpaired student's t-test. IFN interferon, GBP2 guanylate-binding protein 2, Ctrl control, IR ionizing radiation, dpi day post IR, FSC forward scatter, PLP platelet-like particle, siGBP2 siRNA of GBP2, GAPDH glyceraldehyde-3-phosphate dehydrogenase

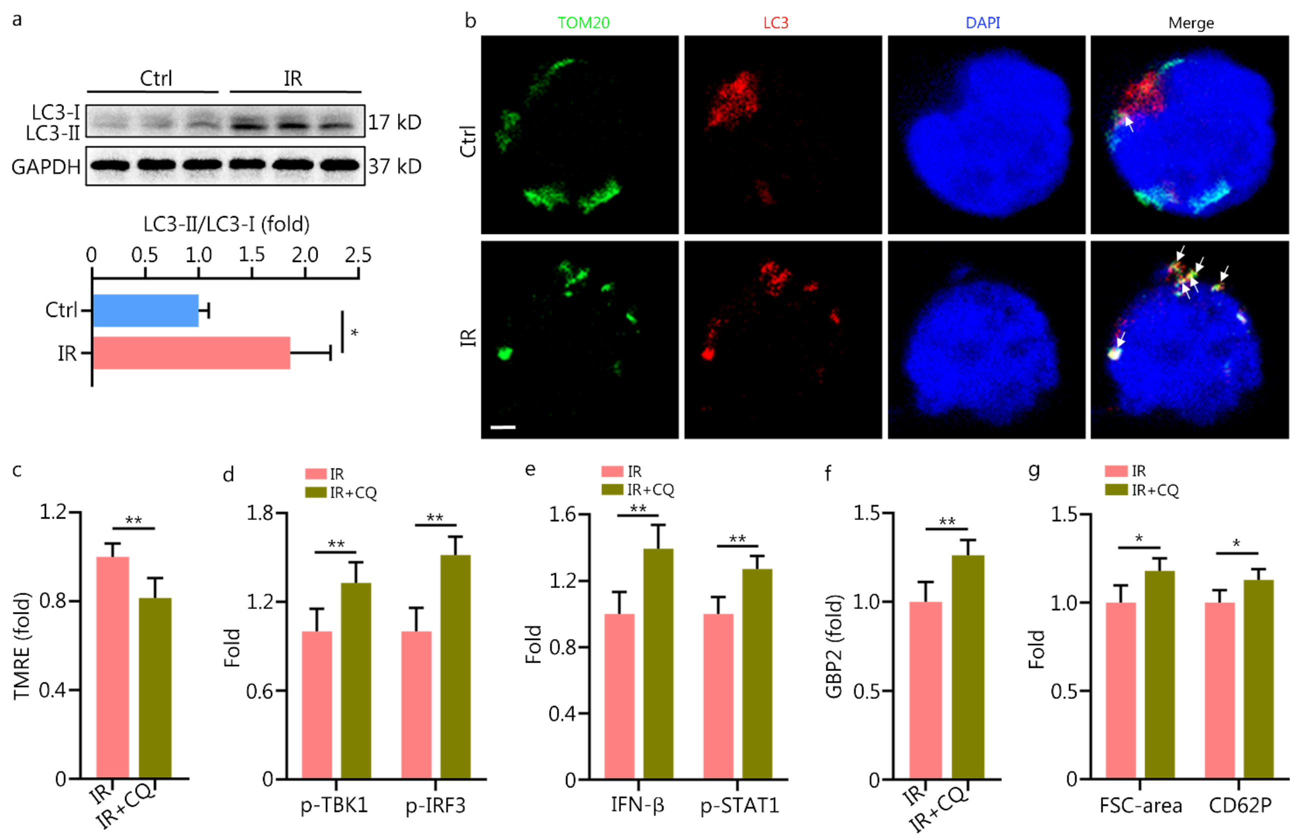

Fig. S8 Autophagy restrains minority MOMP in Meg-01 post IR. **a** Western blotting analysis and quantification of LC3 lipidation in Meg-01 cells at 1 dpi (n = 3). **b** Autophagic clearance of mitochondria in Meg-01 cells at 1 dpi was assessed by immunofluorescence staining with antibodies specific for LC3 and TOM20. Scale bar = 20 μm. **c** Flow cytometric quantification of MMP in Meg-01 cells with or without CQ treatment at 1 dpi (n = 5). **d** Flow cytometric quantification of p-TBK1 and p-IRF3 expression in Meg-01 cells with or without CQ treatment at 1 dpi (n = 5). **e** Flow cytometric quantification of IFN-β and p-STAT1 expression in Meg-01 cells with or without CQ treatment at 1 dpi (n = 5). **f** Flow cytometric quantification of GBP2 expression in Meg-01 cells with or without CQ treatment at 3 dpi (n = 5). **g** Flow cytometric quantification of FSC-area and CD62P expression in response to thrombin of PLPs from Meg-01 cultures with or without CQ treatment at 3 dpi (n = 5). Data represent mean ± standard deviation. $^{*}P < 0.05$, $^{**}P < 0.01$, compared to Ctrl or IR. Two-tailed unpaired student's t-test. MOMP mitochondrial outer membrane permeabilization, Ctrl control, IR ionizing radiation, dpi day post IR, LC3 microtubule-associated protein 1 light chain 3, LC3-I non-lipidized LC3, LC3-II lipidized LC3, TOM20 translocase of outer mitochondrial membrane 20, MMP mitochondrial membrane potential, CQ chloroquine, TBK1 TANK-binding kinase 1, IRF3 interferon regulatory factor 3, IFN-β interferon-β, STAT1 signal transducer and activator of transcription 1, GBP2 guanylate-binding protein 2, FSC forward scatter, PLP platelet-like particle, TMRE tetramethylrhodamine ethyl ester, DAPI 4',6-diamidino-2-phenylindole, GAPDH glyceraldehyde-3-phosphate dehydrogenase
